# Design of SnO_2_:Ni,Ir Nanoparticulate Photoelectrodes for Efficient Photoelectrochemical Water Splitting

**DOI:** 10.3390/nano12030453

**Published:** 2022-01-28

**Authors:** Mohamed Shaban, Abdullah Almohammedi, Rana Saad, Adel M. El Sayed

**Affiliations:** 1 Department of Physics, Faculty of Science, Islamic University of Madinah, Al-Madinah Al-Munawarah 42351, Saudi Arabia; ard.almohammedi@hotmail.com; 2 Nanophotonics and Applications (NPA) Lab, Physics Department, Faculty of Science, Beni-Suef University, Beni-Suef 62514, Egypt; ranasaad811@gmail.com; 3 Department of Physics, Faculty of Science, Fayoum University, El-Fayoum 63514, Egypt; adel_sayed_2020@yahoo.com

**Keywords:** SnO_2_ nanoparticulate thin films, Ni/Ir-doping, photoelectrocatalyst, photoelectrochemical hydrogen generation, conversion efficiencies

## Abstract

Currently, hydrogen generation via photocatalytic water splitting using semiconductors is regarded as a simple environmental solution to energy challenges. This paper discusses the effects of the doping of noble metals, Ir (3.0 at.%) and Ni (1.5–4.5 at.%), on the structure, morphology, optical properties, and photoelectrochemical performance of sol-gel-produced SnO_2_ thin films. The incorporation of Ir and Ni influences the position of the peaks and the lattice characteristics of the tetragonal polycrystalline SnO_2_ films. The films have a homogeneous, compact, and crack-free nanoparticulate morphology. As the doping level is increased, the grain size shrinks, and the films have a high proclivity for forming Sn–OH bonds. The optical bandgap of the un-doped film is 3.5 eV, which fluctuates depending on the doping elements and their ratios to 2.7 eV for the 3.0% Ni-doped SnO_2_:Ir Photoelectrochemical (PEC) electrode. This electrode produces the highest photocurrent density (*J_ph_* = 46.38 mA/cm^2^) and PEC hydrogen production rate (52.22 mmol h^−1^cm^−2^ at −1V), with an Incident-Photon-to-Current Efficiency (IPCE% )of 17.43% at 307 nm. The applied bias photon-to-current efficiency (ABPE) of this electrode is 1.038% at −0.839 V, with an offset of 0.391% at 0 V and 307 nm. These are the highest reported values for SnO_2_-based PEC catalysts. The electrolyte type influences the *J_ph_* values of photoelectrodes in the order *J_ph_*(HCl) > *J_ph_*(NaOH) > *J_ph_*(Na_2_SO_4_). After 12 runs of reusability at −1 V, the optimized photoelectrode shows high stability and retains about 94.95% of its initial PEC performance, with a corrosion rate of 5.46 nm/year. This research provides a novel doping technique for the development of a highly active SnO_2_-based photoelectrocatalyst for solar light-driven hydrogen fuel generation.

## 1. Introduction

Photoelectrochemical water splitting (PEC-WS) and the hydrogen generation process using unlimited solar energy have attracted increased attention worldwide. Hydrogen energy is extensively used in various fields, such as rocket propellant, vehicle fuel, and refining petroleum products. This is due to its zero CO_2_ emissions, low density, high energy density, environmental friendliness, renewability, and storage capacity. This technique requires a large-area photoelectrode and a low-cost chemically stable material with significant absorption of solar light and a suitable bandgap [1,2,3,4]. 

Demonstrating abundance, facile preparation, chemical stability in a wide range of pH values [1], and environmental compatibility [5], SnO_2_ is a semiconductor material that inherits a direct bandgap (*E*_g_) of 3.6 eV with a high exciton binding energy at room temperature (RT) [2]; its electron mobility is in the order of 240 cm^2^ V^−1^ S^−1^ [6], and it has high transparency in the visible range, low resistance, and high reflectivity for infrared light [7]. This oxide’s characteristics make it ideal for optoelectronics, solar cells, gas sensors, catalysis, and thin-film transistors (TFTs). Sn^4+^ (4d^10^5s^0^) is suggested to replace In^3+^ in thin-film transistors to avoid the high cost of device fabrication [8]. On the other hand, and despite these many advantages of SnO_2_, its wide *E*_g_ and low surface area limit its use in industrial applications such as hydrogen generation.

Various physical and chemical approaches have been reported for the fabrication of doped and pure SnO_2_ films and nanopowders. Sb-doped SnO_2_ films were synthesized by means of the dip-coating technique [7] and spray pyrolysis [9] for possible use in optoelectronic applications and photocurrent generation. A SnO_2_/ graphene sheet prepared by means of a hydrothermal method showed photocatalytic activity in dye removal, and this composite was found to be suitable as a supercapacitor electrode [10]. Bera et al. [11] synthesized uniform SnO_2_ films of nanograss structure and SnO_2_/BiVO_4_ core-shell structures by means of a vapor-solid deposition process to achieve high photoelectrochemical efficiency. Moreover, Mohammad et al. [5] fabricated SnO_2_-g-C_3_N_4_ nanostructures by means of the hydrothermal route for dye removal and wastewater treatment. Yuan et al. [12] found that WO_3_@SnO_2_ core-shell nanosheets with a 20 nm SnO_2_ shell layer prepared using atomic layer deposition have superior sensing performance with a response of 1.55 and selectivity toward 15 ppm NH_3_ at 473 K.

Among the used elements, the transition metals represent a fascinating class of materials as dopants. Doping with Co was reported to enhance the hydrogen gas sensing properties of the spin-coated SnO_2_ films [3]. Cr doping was applied to tune the linear and nonlinear optical parameters of the spray-deposited SnO_2_ thin films [13]. Moreover, doping SnO_2_ films with Fe (5–20%) caused the transition of the conductivity of SnO_2_ from n- to p-type and induced a good rectifying character for SnO_2_ homojunction [14]. Ni doping has been reported to induce a greater number of defects and oxygen vacancies, resulting in an RT ferromagnetic character for the co-precipitated SnO_2_ nanoparticles (NPs) [15]. Additionally, the Ni-doped SnO_2_ was extensively studied for gas sensing applications [16,17]. Additionally, co-doping or double doping seems to be required for tuning the materials’ properties. Saadeddin et al. [18] found that the double doping of SnO_2_ by Zn and Sb significantly improved its electrical conductivity and density, and the presence of Zn prevents the Sb evaporation during solid-state reaction at 1300 °C. According to Lamrani [19], the double (Eu, Gd) impurities substitute the adjacent Sn sites and result in strong ferromagnetic interactions via the *p*–*f* hybridization between rare-earth 4f and Op states. The obtained ferromagnetic alloy is expected to act as a great power source for effective photovoltaic conversion in solar cell applications. Moreover, Sery et al. [20] proved that co-doping of SnO_2_ NPs with (3% Ti, 3% Ni) and co-doping of NiO NPs with (3% Ti, 3% Sn) significantly reduced their *E*_g_ and the obtained oxides are highly efficient photocatalysts. 

The present work was focused on evaluating the influence of two transition metals, the noble metals Ir and Ni, on the physicochemical features of SnO_2_ material. This work represents the first attempt to improve the structural, optical, and photoelectrochemical characterization of SnO_2_:Ni,Ir films to enhance their potential for hydrogen energy generation. The samples were prepared by means of a sol-gel method, owing to its low cost, simplicity, safe, mass/large-scale production, and easy tailoring of film thickness. Additionally, sol-gel-prepared films usually have a mesoporous structure or a high specific area, which made these films more suitable for gas sensing and hydrogen energy production [3].

## 2. Experimental Details

### 2.1. Chemicals and Preparation Process

The source of Sn was SnCl_2_·2H_2_O, MW~225.63, from Merck, whereas the source of Ir element was IrCl_3_·H_2_O, MW~298.58 g/mol from Sigma. The Ni source was Ni(CH_3_COO)_2_. 4H_2_O, MW ~ 248.84 from Fairsky. High-purity and water-free ethanol was used as a common solvent, and glacial acetic acid was used as a stabilizer. Meanwhile, 0.325 M solutions of SnO_2_ and 3.0% Ir-doped SnO_2_ (SnO_2_:Ir) were synthesized by dissolving the required amounts of SnCl_2_·2H_2_O and IrCl_3_·H_2_O/SnCl_2_·2H_2_O, respectively, in 10 mL ethanol for each. These two solutions were then stirred at 60 °C for 3 h. After the first 20 min, a predetermined amount of the stabilizing agent was added drop by drop to obtain a clear and homogeneous solution. Then, the solutions were aged for one day at RT. To obtain SnO_2_:Ni,Ir films, Ni(CH_3_COO)_2_·4H_2_O was added to the second solution with specific weighted amounts for 1.5%, 3.0%, and 4.5% Ni-doped SnO_2_:Ir structures. The spin coating process was carried out on pre-cleaned and dried glass substrates, and at 2500 rpm for 25 s, followed by drying for 15 min at 200 °C. The coating and drying processes were repeated 7 times. Finally, the films were subjected to heat treatments at 450 °C for 3.0 h in a controlled air furnace followed by cooling the furnace to RT overnight. 

### 2.2. Characterization and Measurements

The crystal structure identification of doped and pure SnO_2_ was evaluated using a high-resolution X-ray diffractometer PANalytical X’Pert Pro system, Holland. This was done using CuKα radiation of wavelength λ = 1.5406 Å with a step of 0.02° and operated at 42 kV and 32 mA. The patterns were recorded in the range of 4–70° on the 2θ-scale. The films’ surface morphology, thickness, and root mean square roughness (R_rms_) were determined by means of atomic force microscope (AFM), the PARK SYSTEM, and XE-100E, respectively. Fourier transform infrared spectroscopy (FTIR) was used to investigate the films’ functional groups, utilizing FT/IR-6200 (Jasco Co., Tokyo, Japan), in a wavelength range 400–4000 cm^−1^. The absorbance spectra (Abs) were recorded in a wavelength range of 200–1000 nm using a UV/VIS/NIR 3700 (Shimadzu, Columbia, MD 21046 USA) double beam Shimadzu spectrophotometer. The absorption coefficient α=2.303 Abs./film thickness (d) was used in calculating the direct optical *E_g_* of the samples. All these investigations were performed at RT. The photoelectrochemical water splitting measurements were performed using 100 mL of 0.5 M HCl, NaOH, or Na_2_SO_4_ solutions at RT (25 °C) with a sweep rate of 1 mV/s. Only the temperature effect was investigated using a 0.1 M HCl solution. In the water-splitting test reaction, a two-electrode cell was used, with the photocathode being a nanocomposite electrode with a 1 cm^2^ surface area and the counter electrode being a Pt electrode. The artificial light was incident on the electrode surface with standard white light illumination of 100 mW·cm^−2^ provided by a 500 W Xenon lamp (Newport, 66142-500HX-R07, Newport, UK). The effects of incident wavelength (307–636 nm), temperature reaction (25–85 °C), and time stability on water splitting were investigated.

## 3. Results and Discussion

### 3.1. X-ray Diffraction and AFM Analysis

**Figure 1:**
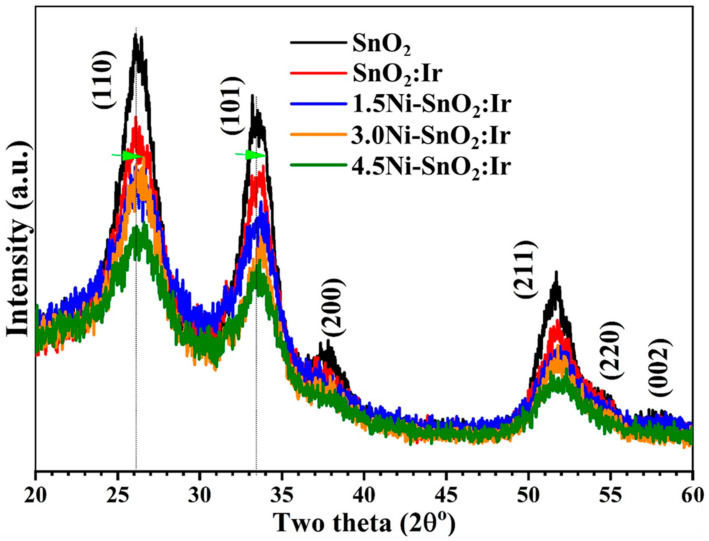
XRD patterns of SnO_2_, 3.0% Ir-doped SnO_2_ and SnO_2_:Ni,Ir thin films. The arrows indicate the peak shift.

Figure 1a shows X-ray diffractometer (XRD) charts of doped and pure films. The patterns are composed of well-defined peaks with orientations in the (1 1 0), (1 0 1), (2 0 0), (2 1 1), (2 2 0), and (0 0 2) planes that match the standard data for a rutile phase of cassiterite SnO_2_ with a tetragonal structure, JCPDS no. 72-1147, and space group P4_2_/mnm. Among them, the (1 1 0) peak exhibits the highest intensity, and this direction has the least surface energy, and so is thermodynamically and electrostatically the most stable [21]. Moreover, the crystal growth in the (2 0 0) and (2 1 1) directions means that all films are polycrystalline and composed of randomly oriented crystals. No IrO_x_ or NiO phases are observed in the spectra under the resolution limit of XRD. A similar result was reported for hydrothermally prepared graphene-loaded SnO_2_ NPs [10]. Figure 1b illustrates that the positions of (1 0 1) and (2 0 0) are influenced by doping, where the peaks are right-shifted with an increasing Ni doping ratio. These peaks’ shift confirms that the Ir/Ni ions were introduced substitutionally and/or interstitially inside the SnO_2_ lattice [22]. Right-shifted diffraction peaks were also noticed in the XRD of spin-coated SnO_2_ after loading with Co [3]. Additionally, the introduction of Zn^2+^ (0.74 Å radius) and F ions (1.33 Å radius) caused lattice expansion for the spray-deposited SnO_2_ films [23]. 

The crystallite size (*D*) and lattice parameters (*a*,*c*) were determined from Scherrer’s equation, $D=0.9\lambda /(\beta_{1/2}\cos\theta )$ , and the following formula [22]:

(1) $$\frac{1}{d_{hkl}}=\frac{2\;sin\theta }{\lambda }=\sqrt{\frac{\left(h^{2}+k^{2}\right)}{a^{2}}+\frac{l^{2}}{c^{2}}}$$

where $\beta_{1/2}$ is the full width at half maximum intensity, *d* is the inter-planar distance, and (hkl) is Miller’s indices. The calculated values of *a*, *c*, and the volume of the unit cell (*V* = *a^2^c*), as well as the crystallite size (*D*_av_), are tabulated in Table 1. As apparent from Figure 1, the peaks’ intensity reduced with an increasing dopant concentration, indicating the decrease in the crystallinity of the films. The value of *D*_av_ was calculated from the most three intense peaks; (1 1 0), (1 0 1), and (2 1 1). *D*_av_ slightly increased from 29.62 to 30.43 nm after 3.0% Ir doping but decreased to 24.23 nm after 4.5% Ni loading. This means that Ir doping improved the film’s crystallinity. The decrease in *D*_av_ of SnO_2_ with increasing dopant contents may be attributed to the formation of a large number of nucleation centers causing the formation of smaller nanocrystals [24]. The greater the density of centers of nucleation, the smaller *D*_av_ will be, and vice versa [25]. Similarly, *D*_av_ decreased from 28.3 nm for pure SnO_2_ nanorods to 6.2 nm after 5.0% Ni doping [16]. As listed in Table 1, the calculated values of *a*, *c*, and V of pure SnO_2_ are 4.747 Å, 3.189 Å, and 71.86 Å^3^, respectively. These values are close to those reported by Bouznit and Henni [9], *a* = 4.75 Å and *c* = 3.18 Å for the spray-deposited SnO_2_ film. Additionally, the parameters of the prepared samples are influenced by the dopant type and its content inside the SnO_2_ film. When increasing the Ni content from 1.5% to 4.5%, the *a*, *c* and V are decreased from 4.741 Å, 3.182 Å, and 71.52 Å^3^ to 4.712 Å, 3.173 Å, and 70.45 Å^3^, respectively. The decrease in these parameters is attributed to the replacement of the Sn^4+^ ion (radius ~0.72 Å) with smaller sized ions (Ir^3+^, 0.68 Å; Ni^2+^, 0.62 Å), as well as the associated interfacial effects at the dopant sites, such as charge redistribution and valence state regulation [26,27]. This also may be related to the nanoparticle size effect, whereas the lattice parameter contraction is proportional to the diameter of the nanoparticle. Singh et al. [2] reported a reduction in *D*_av_ and an increase in V of the co-precipitated SnO_2_ NPs due to doping with Er at 1.0–5.0% ratios. 

**Figure 2:**
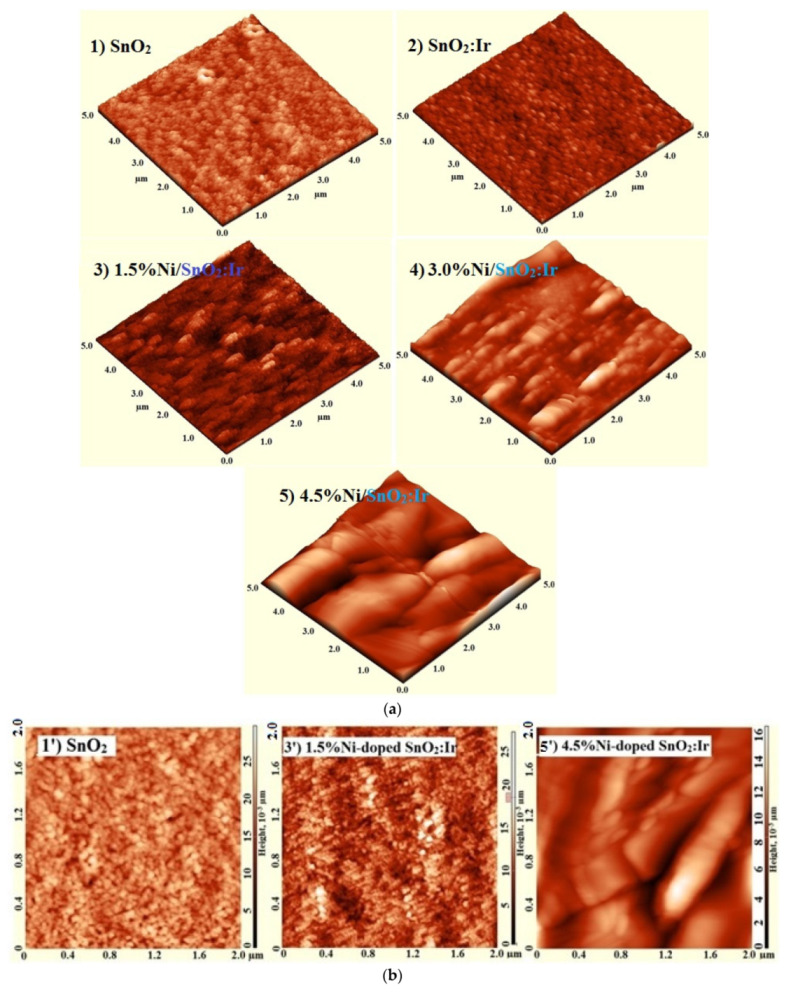
(**a**) Three-dimensional AFM images for the sol spin-coated SnO_2_, IrSnO_2_, and Ni-doped IrSnO_2_ films. (**b**) Two-dimensional AFM images for the SnO_2_, 1.5% and 4.5% Ni-doped SnO_2_:Ir films.

Figure 2 shows 3D and 2D AFM images for the doped and pure SnO_2_ films. As noted, the substrates are well-covered with the material, and films are homogeneous, compact, and crack-free with a granular or particulate character. The surface morphology slightly changed after Ir loading, but depends on the Ni doping level. The white spots are nanocrystal islands with heights exceeding 6 nm in SnO_2_ film and 10 nm in the Ni-doped IrSnO_2_ films. Notably, 3.0% Ni loading changes the surface of SnO_2_:Ir to a 1D tubular nanostructure, which is attractive for solar conversion via offering direct pathways for the charge carrier transport [6]. The films’ thickness is in the range of 215–225 nm, as listed in Table 1, i.e., the average of this range is ~220 nm. The grain size (Gs) and root mean square roughness (R_rms_) of all films are listed in Table 1. The Gs marginally increased after introducing Ir^3+^, due to its comparable radius with that of the host (Sn^4+^). Then, the Gs decreases from 35.07 nm for SnO_2_:Ir film to 23.81 nm with an increasing Ni doping ratio from 0 to 4.5%. The difference in radii between host Sn^4+^ and dopant Ni^2+^ may account for the decrease in Gs caused by increasing the Ni doping level. This behavior is consistent with the data obtained from XRD analysis, peak shift, lattice parameters, and unit cell volume. Previously, it was mentioned in [17] that Ni is a grain growth inhibitor within the SnO_2_ matrix. This result is consistent with XRD data. However, as AFM measures Gs as the apparent distance between two adjacent grains, and as one grain may consist of more than one crystallite, Gs is thus found to be greater than *D*_av_. Moreover, the R_rms_ of SnO_2_ decreased from 17.25 to 15.36 nm after doping with 3.0% Ir, then increased to 19.17 nm after co-doping with 1.5% Ni. Finally, it decreased to 11.64 nm with an increased Ni doping ratio. Similar results were reported for the sol-gel dip-coated (Fe, Ni)-doped SnO_2_ films [28]. In that paper, Benkara et al. illustrated that the R_rms_ strongly depends on the preparation and dipping conditions as well as the different kinetics of Fe and Ni needed for the growth of SnO_2_ nanoparticles. It is well-known that low R_rms_ reduces the carrier scattering inside the films, ensuring the high performance of TFTs [8]. Additional morphological investigation was carried out by taking cross-sectional images for the films, as shown in Figure S1 (Supplementary Materials). These images confirm the tubular structures of the prepared films after the insertion of Ni and Ir as dopant materials. Surface morphology, it is believed, plays an important role in improving the material’s PEC performance. As a result, Ni and Ir doping is expected to encourage the use of these films as photoanodes in electrochemical applications.

### 3.2. FTIR and UV-Vis Spectroscopy

**Figure 3:**
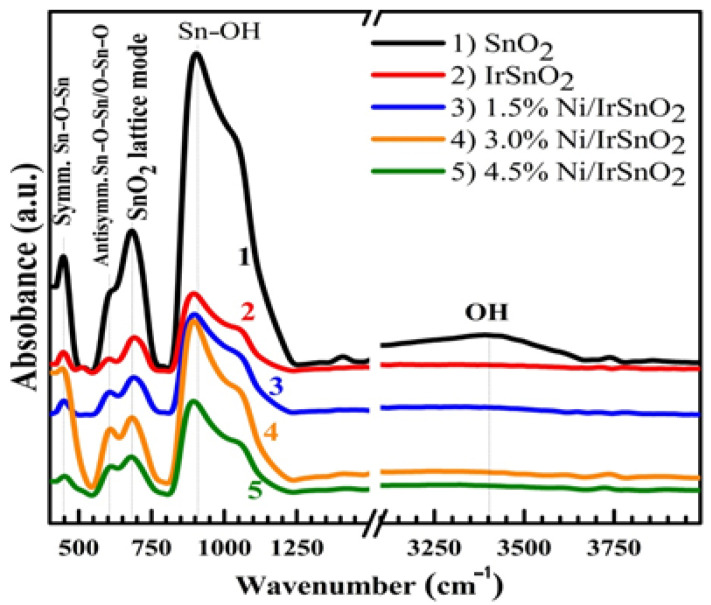
FTIR absorption spectra of SnO_2_, 3.0% Ir-doped SnO_2_ (SnO_2_:Ir) and SnO_2_:Ni,Ir.

In the absorbance mode, the FTIR spectra of SnO_2_, SnO_2_:Ir, and SnO_2_:Ni,Ir samples are displayed in Figure 3. The absorption band at 445 cm^−1^ is owing to the symmetric Sn–O–Sn vibration. The band at 604 cm^−1^ is attributed to the antisymmetric Sn–O–Sn/O–Sn–O bridge groups of SnO_2_ [26]. Doping with Ni/Ir results in a slight decrease for the first band and a slight increase for the second one. The SnO_2_ lattice mode appears at 688 cm^−1^, and this band is also influenced by foreign atoms [22]. The observed bands are similar to those reported by Kuantama et al. [29] for nanoporous F: SnO_2_ prepared by the sol-gel combustion route. The very strong infra-red (IR) absorption that occurs at ~905 cm^−1^ was assigned to Sn–OH vibration [30]. Besides the reduction in intensity with doping, this band is also slightly shifted to lower energies. Moreover, the IR spectrum of SnO_2_ film displays a broad peak around 3410 cm^−1^, indicating the presence of O–H on the film surface. The IR absorption spectrum is size- and morphology-dependent [28]. The film doped with 3.0% Ni shows more intensive absorption bands compared with the films doped with 1.5 and 4.5% Ni. This is due to the small crystallites size at this doping ratio (23.91 nm) and the change to one-dimensional (ID) tubular morphology, as seen in Figure 2. The observed red-shift and decrease in peaks’ intensity due to the Ni/Ir doping are powerful pieces of evidence of the Ir and Ni introducing into the SnO_2_ cassiterite lattice. The observed peaks related to the presence of OH indicate the films’ ability to be used in aqueous solutions for electrochemical applications.

**Figure 4:**
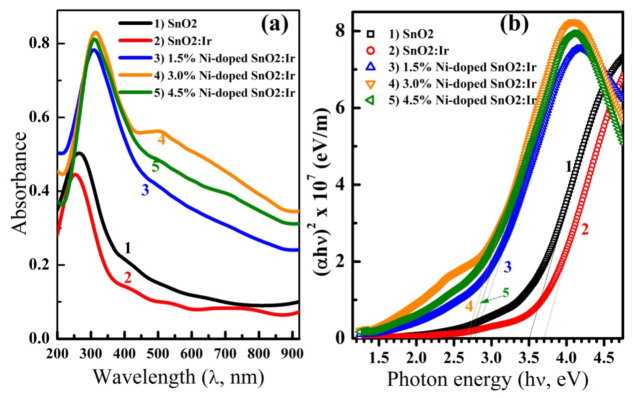
(**a**) UV-vis absorption spectra and (**b**) the bandgap energy determination of SnO_2_, SnO_2_:Ir and 1.5%, 3.0% and 4.5% Ni-doped SnO_2_:Ir.

UV-vis spectra of the films are shown in Figure 4a. As seen, although Ir incorporation reduced Abs, there is a significant increase in Abs after introducing Ni into the IrSnO_2_ matrix. Ni-doped films show an absorption band around 255 nm, shifted to lower wavelengths for SnO_2_ and SnO_2_:Ir films. This band is due to the photoexcitation of electrons from the valence band (VB) to the conduction band (CB) [15]. Moreover, the absorption edge is shifted from 380 nm for the pure film to longer wavelengths after doping with Ni. Similarly, compared to the electro-spun SnO_2_ nanotubes (NTs), the SnO_2_/SnS_2_ showed an absorption edge that red-shifted combined with an enhanced absorption intensity [31]. Since visible light represents a large portion of the solar spectrum, photoanodes with strong absorption for the visible light (i.e., Ni-doped films) should have higher PEC-WS performance. 

Figure 4b shows (αhν)^2^ vs. hν, the Tauc plots for determination of *E*_g_, which are obtained by linear extrapolation to the abscissa axis. As given in Table 1, the *E*_g_ of pure SnO_2_ is ~3.5 eV, and 3.0% Ir incorporation resulted in a blue-shift for *E*_g_ to 3.7 eV, whereas a red-shift to 2.70 eV is caused by Ni loading. This is consistent with the introduction of electronic states in the bulk and the oxygen defects associated with the doping. Although the *E*_g_ of the pure film is smaller than that reported in the literature, Mohammad et al. [5] reported an *E*_g_ of ~3.19 eV for the hydrothermally prepared SnO_2_ film, and Li et al. [31] reported an *E*_g_ of ~3.37 eV for SnO_2_ nanotubes fabricated by the electrospinning technique. The Burstein–Moss effect could explain the widening in *E_g_* due to Ir doping. The narrowing in *E*_g_ with Ni doping may be related to the random distribution of Ni impurities and formation of the density of states tails at the edges of the bands, which result in an increased number of band-to-tail and tail-to-tail transitions [23]. In other words, this is due to the *sp*-*d* spin-exchange interactions between the localized *d* electrons of Ni ion substituting the cation and the band electrons. This result suggests that Ir/Ni co-doping could be used to tune the *Eg* of SnO_2_ for specific applications.

In the literature, the *E*_g_ of SnO_2_ reduced from 3.87 to 3.79 eV after doping with 10% Sb [7] and decreased from 3.6 to 3.28 eV after doping with Cr at ratios ≤ 5 wt% [13]. Moreover, according to Othmen et al. [14], the incorporation of Fe (5–20%) decreased the allowed and forbidden direct *E*_g_ of spin-coated SnO_2_. Ni doping can alter the *E*_g_ of SnO_2_ NPs from 4 to 3.85 eV [15]. Additionally, Ni doping at 5.0% reduced the *E*_g_ of SnO_2_ nanorods, prepared by a hydrothermal method, from 3.85 to 3.65 eV [16]. According to Salameh et al. [23], the *E*_g_ of SnO_2_ decreased from 3.97 to 3.53 eV after Zn loading but increased to 4.45 eV after co-doping with F. Han et al. were also able to reduce the energy gap of sol-gel-prepared SnO_2_ films from 3.8 to 3.1 eV by using Ti alloying and the oxygen vacancies created [32]. In contrast to our results, Ren et al. [8] found that the *E*_g_ of SnO_2_ was 3.87 eV and was not changed even after doping with 10–50% Ga. Additionally, Ni doping was found to increase the *E*_g_ of the spray-deposited SnO_2_ films from 4.03 to 4.09 eV [17]. Guo et al. obtained SnO_2_ electrode with an energy gap of 1.74 V after (Ni, S)-co-doping [33]. Additionally, Matussin et al. reduced the band gap energy from 3.33 eV for SnO_2_ to 2.08 eV for 10at% (Co, Ni)- SnO_2_ [34]. Moreover, Jasim et al. reported the reduction in the *E_g_* of SnO_2_ from 3.7 to 2.5 eV for SnO_2_:Co: Cu–H nanoparticles [35] 

### 3.3. Photoelectrochemical H_2_ Generation

#### 3.3.1. Influence of the Used Electrolyte

**Figure 5:**
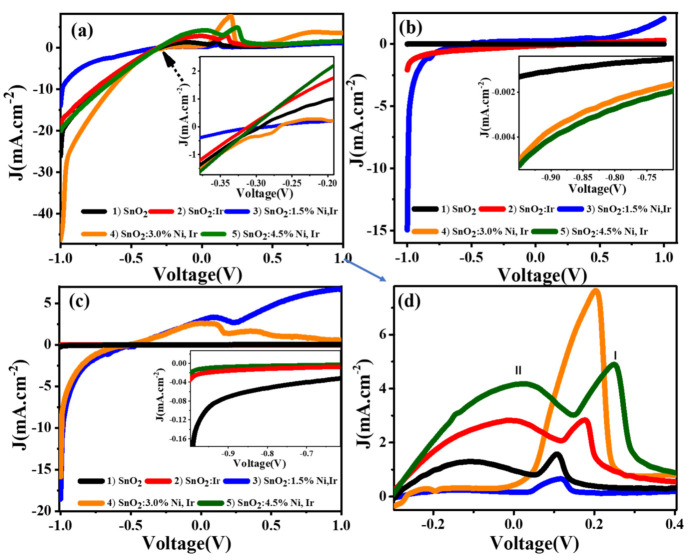
Electrodes’ photocurrent density–voltage curves in 100 mL of 0.5 M (**a**) HCl; (**b**) Na_2_SO_4_; (**c**) NaOH; and (**d**) anodic current density versus voltage of peak I and peak II. The insets of (**a**–**c**) represent enlarged parts of the photocurrent density–voltage curves.

One of the significant parameters that affect the PEC performance of the photocatalyst is the PEC cell electrolyte. Any alteration in the conditions of the electrolytes will cause several differences in the photocatalytic interfaces. Electrolyte choices need to be consistent with the applied PEC catalyst. Electrolytes must not have electrochemical reactions with the used photocatalysts, and their optical absorption bands cannot overlay with that of photocatalysts. The structure of electrolytes has a major influence on the PEC H_2_ generation rate, which affects the diffusion of ions and thus changes the conductivity of electrolytes. The H_2_ generation rate and PEC catalytic efficiency depend on the pH values and the conductivity of the electrolytes [36]. Conductivity is an essential property for selecting electrolytes to allow the transport of charges in the PEC process. The addition of active cations/anions (such as Na+/SO_4_^2−^ or OH^−^) with sufficient concentrations will reduce the electrolyte impedance and promotes band bending. Dissimilar electrolytes of the same concentration (0.5 M)—a strong acid (HCl, pH = 0.3), a strong base (NaOH, pH = 13.69), and neutral salt (Na_2_SO_4_)—were employed to identify the major variations in the PEC performances of the studied photocathodes. The PEC measurements of the photoelectrodes were taken in 100 mL of each electrolyte at 25 °C (RT) with increment 1 mV/s. During PEC measurements, a working 1 cm^2^ electrode and a counter 1 cm^2^ Pt electrode were used. The measurement of photocurrent density–voltage (*J_ph_*–V) curves was performed in standard white light illumination of 100 mW.cm^−2^ provided by a 500 W Xenon lamp, as shown in Figure 5a–c. The *J_ph_*’s value rose as the used bias increased and, surprisingly, the current density increased following the order: *J_ph_*(HCl) > *J_ph_*(NaOH) > *J_ph_*(Na_2_SO_4_). The maximum *J_ph_* value is 46.38 mA/cm^2^ in HCl, 18.54 mA/cm^2^ in NaOH, and 14.95 mA/cm^2^ in Na_2_SO_4_ solution. So, HCl is the best electrolyte for the PEC process utilizing the 3.0% Ni-doped SnO_2_:Ir photoelectrode. Similar behavior was reported for F-doped and In-doped SnO_2_ [37]. This could be due to a slightly low pH value (0.30) that enhances the PEC reaction in an acidic medium with a high density of H^+^ ions [38]. The obtained *J_ph_* value for 3.0% Ni-doped SnO_2_:Ir photoelectrode is higher than that obtained for 1.5% Ni-doped SnO_2_:Ir, 4.5% Ni-doped SnO_2_:Ir, SnO_2_:Ir, and SnO_2_ under exposure to light. So, the optimized photocathode is 3.0% Ni-doped SnO_2_:Ir, which gives the maximum photocurrent density in the HCl electrolyte.

On the other hand, the observed anodic current especially in the HCl electrolyte (Figure 5d) is ascribed to the oxygen and chlorine evolution reactions (OER and CER) in the HCl electrolyte (Peak I and Peak II) for SnO_2_ and SnO_2_:Ir electrodes and only OER (Peak I) for 1.5% Ni-doped SnO_2_:Ir and 3.0% Ni-doped SnO_2_:Ir electrodes. The current density and voltage position of peaks I and II are shown in Table S1 (Supplementary Materials). For Na_2_SO_4_ (Figure 5b), the anodic current at a voltage > 0.5 V is ascribed to the OER only. It is noted that the use of NaOH (Figure 5c) decreased the OER efficiency of the SnO_2_:3% Ni,Ir sample, which was found to occur because OH^−^ ions fight for adsorption sites and the kinetics of the reaction may be deaccelerated. Therefore, in the different electrolytes, the recorded anodic currents were predominantly due to oxygen evolution depending on the electrolyte, and not to SnO_2_:Ni,Ir dissolution. Hence, in the various electrolytes, the observed anodic currents were mainly caused by oxygen evolution depending on the electrolyte, and not SnO_2_:Ni,Ir dissolution [39]. So, under OER conditions, Ni and Ir co-doping could help to improve PEC stability, as we will discuss later. It was previously stated that the band bending at a high doping level increased with anodic current density, resulting in a monotonic current density increase at pH ≤ 1 due to the driven electron flow through the band bending and ionization of the deep donor levels of oxygen vacancies [40]. Electron tunneling is considered the principal mechanism of anodic current density at low doping percentages [41]. 

#### 3.3.2. PEC Behavior of the 3.0% Ni-Doped SnO_2_:Ir Photocathode

**Figure 6:**
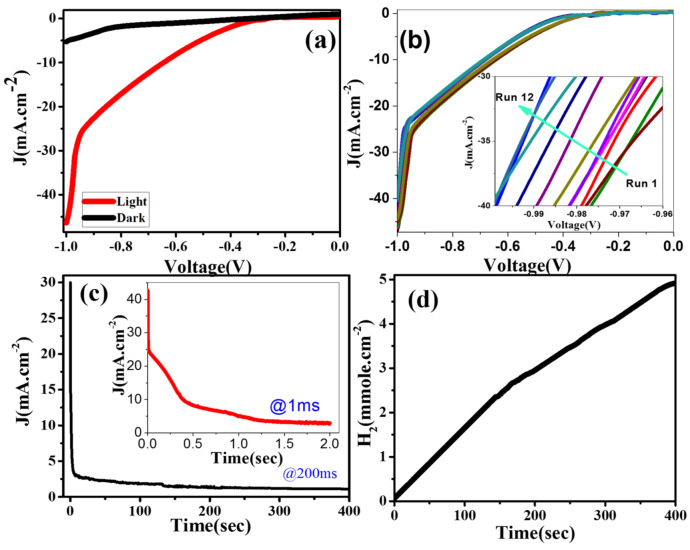
The dependence of *J_ph_* on the applied voltage of the 3.0% Ni-doped SnO_2_:Ir photoelectrode (**a**) in the dark and under white light and (**b**) for 12 runs of reusability under white light illumination at room temperature; (**c**) *J_ph_* versus exposure time and (**d**) the number of H_2_ moles at time of generation for the 3.0% Ni-doped SnO_2_:Ir photocathode at −1V and room temperature. The insets of (**b**,**c**) showed magnified parts of the curves.

Figure 6a shows the PEC characteristics of the 3.0% Ni-doped SnO_2_:Ir photoelectrode utilizing 0.5M HCl in the dark and under the standard white light of 0.1 W/cm^2^ illuminations utilizing a Keithley 2400 source measure unit. As shown, the current density increased with an increasing voltage to reach −5.27 and −46.38 mA cm^−2^ at −1V in the dark and when subjected to white light, respectively. The significant dark currents are caused by the transfer of charges supported by ionic currents that come from the HCl electrolyte. Under the visible light illumination, the sharp increase in *J_ph_* with a negative potential indicates that this electrode acts as a photocathode. Remarkably, this PEC electrode displays a current density of ∼−432 μA/cm^2^ at zero V and photocurrent onset at >−0.196 V. The high value of the photogenerated current density in light is a pre-indicator for the high-performance PEC water-splitting. This could be due to the extension of *E_g_* to the visible light region (2.7 eV) and the robust absorption of Vis/IR by Ni and Ir co-doping, in addition to the observed 1D nanotubular morphology, which speeds up the redox reaction rate and thus promotes the PEC reaction in addition to enhancing the tunneling activity of the photogenerated carriers [42,43]. 

The analysis of the PEC (*J_ph_*–V) curve was performed under visible light for 12 runs to investigate its ability to be reproduced and the reusability of 3.0% Ni-doped SnO_2_:Ir photoelectrode. These results are shown in Figure 6b. The successive repeated measurements led to a decrement in the photocurrent density from −46.38 to 44.04 mA cm^−2^ after 12 sequent runs at −1 V in 0.5M HCl. Therefore, after 12 runs of the ruse, this electrode conserves almost 94.95% of its initial PEC performance, which indicates high stability and reusability. The PEC stability of the 3.0% Ni-doped SnO_2_:Ir photocathode was studied for a long time in 0.5M HCl under standard visible light illumination and −1V between the Pt counter electrode and this photocathode. The dependence of J on time is depicted in Figure 6c. As seen, the *J_ph_* sharply decreased within the first 2 s to ~3.23 mA·cm^−2^. This rapid fall in *J_ph_* could be due to limited photocorrosion occurring between the redox electrolyte and this photoelectrode [43]. After that, a small decrease in *J_ph_* is seen before reaching −1.28 mA cm^−2^, as a constant value, for a time of >300 s, i.e., the current density decreases until a minimum is reached, illustrating the continuous accumulation of uncompensated ionic space charge at the two electrodes until beginning the injection of electronic charge. This shows that the 3.0% Ni-doped SnO_2_:Ir is photochemically stable and has a long service life for H_2_ generation through the PEC water splitting. Moreover, the photocurrent density was measured under pulsed light utilizing the chronoamperometry technique. 

**Figure 7:**
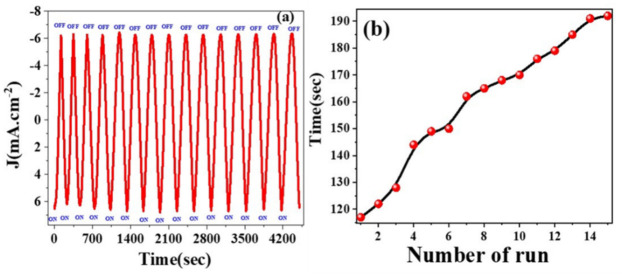
(**a**) Photocurrent intensity for of 3.0% Ni-doped SnO_2_:Ir photocathode under successive on/off illumination cycles and (**b**) time versus number of runs, measured in 0.5M HCl electrolyte under a bias potential of −0.53 V.

Figure 7a shows the variability in the photocurrent density versus the elapsed time during the on/off light switching for the 3.0% Ni-doped SnO_2_:Ir electrode. Figure 7a shows the time required to reach a current density of 6.3 mA/cm^2^ for 15 successive on/off cycles of light. This current/time behavior was measured at −0.53 V. Additionally, the variation of the detection time with the number of runs is provided in Figure 7b. For runs from 1 to 15, the time required to reach 6.3 mA/cm^2^ increased from 117 to 192 s. The photocurrent density decreases in the first two cycles and then reaches a steady and quasi-reproducible state after several on/off cycles of light, with no over-shooting at the beginning or end of the on/off cycle, as shown in Figure S2 (Supplementary Materials). The duration of Figure S2 is 35 s, which represents the electrode’s holding time in the light. This suggests that the electron’s diffusion path is free from grain boundaries, which can introduce traps to impede electron movement and slow down the photocurrent generation [42]. The stability test of the 3.0% Ni-doped SnO_2_:Ir electrode under successive on/off illumination cycles was carried out for 5 h to ensure long-term PEC stability, as shown in Figure S3 (Supplementary Materials).

Theoretically, the calculated number of generated hydrogen moles from the water splitting through the PEC process using Faraday’s law is represented by Equation (2) [44]:

(2) $$H_{2}(\mathrm{moles})=\;\int_{0}^{t}\frac{J_{ph}\mathrm{dt}}{F}$$

where *F* = 9.65 × 10^4^ C/mol (the Faraday constant) and *t* is the time of generation. From the recorded *J_ph_*–time data (Figure 6c), the number of produced H_2_ moles as a function of the time is presented in Figure 6d. The calculated hydrogen output rate for the 3.0% Ni/IrSnO_2_ electrode is 52.22 mmol h^−1^cm^−2^, while practically it was 35.03 mmol h^−1^cm^−2^. The Faraday efficiency is thus 67.1% for 3.0% Ni/IrSnO_2_. 

#### 3.3.3. Effect of Temperature and Thermodynamic Paramters

**Figure 8:**
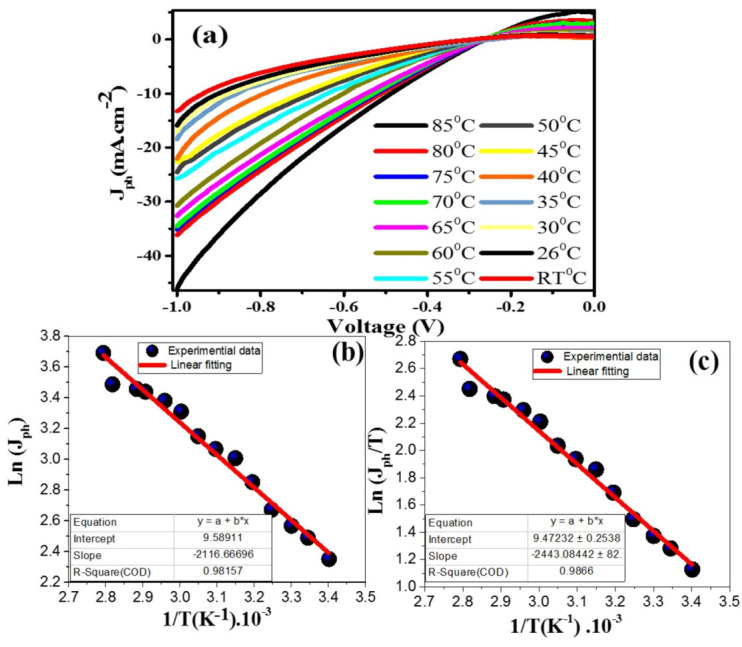
Effect of temperature: (**a**) the *J_ph_*-voltage plots at temperatures in the range from RT to 85 °C, (**b**) ln(*J_ph_*) and (1/T) plots, and (**c**) Ln (*J_ph_*/T) and (1/T) plots.

Figure 8a illustrates the influence of heating in the RT–85 °C range on the PEC J_ph_–voltage of the 3.0% Ni-doped SnO_2_:Ir photoelectrode in 50 mL of 0.1M HCl electrolyte. One can see in this figure that *J_ph_* is increased from −13.29 to −46.07 mA cm^−2^ by increasing T to 85 °C. The increase in *J_ph_* with the increase in T may be due to: (i) increasing T of the photo-induced carriers enhancing the number of electrons in the CB and holes in the VB, thus improving the rate of redox reactions and *J_ph_*; (ii) increasing T leading to an increase in the charge carrier’s mobility and thus the carrier’s lifetime, following the relation μ= q $\tau_{n}$ /m*, where μ indicates the carrier’s mobility, and m* is the carrier’s effective mass. This results in a decrement in the rate of recombination of the produced carriers, which increases the rate of hydrogen generation; and (iii) the improvement of the minority carrier diffusion length and hence the photocurrent density, which is directly proportional to the square root of the absolute T according to the relation *J_ph_* α L_diff_ = $\sqrt{\mu \;\frac{k_{B}T\;}{q}{\;\tau }_{n\;}}$ [45]. 

In addition, it is necessary to evaluate thermodynamic parameters such as activation energy (Ea), enthalpy (ΔH*), and entropy (Δs*). The relationship between the reciprocal of the absolute T (1/T) and *J_ph_* (rate of reaction) for the 3.0% Ni/IrSnO_2_ electrode is depicted in Figure 8b. The Ea value is calculated from the linear fitting slope of Figure 8b based on the Arrhenius equation [46]:

(3) $$\mathrm{Ln}\;\left(J_{ph}\right)=-\frac{\mathrm{Ea}}{R}[\frac{1}{T}]$$

where R = 8.314 J.K^−1^.mol^−1^, the universal gas constant. From Figure 8b, slope = −Ea/R and the Ea value for the 3.0% Ni/IrSnO_2_ electrode is 17.598 kJ.mol^−1^. Additionally, the values of ΔH* and ΔS* for the H_2_ generation reaction are calculated based on the Eyring equation. The Ln (*J_ph_*/T) versus (1/T) is displayed in Figure 8c. This equation takes the form [46]:

(4) $$\mathrm{Ln}\frac{J_{ph}}{T}=-\frac{{\Delta H}^{*}}{R}\cdot \;\frac{1}{T}+\mathrm{Ln}\left(\frac{k_{B}}{h}\right)+\frac{{\Delta S}^{*}}{R}$$

where *k_B_* = 1.38 × 10^−23^ J.K^−1^ (Boltzmann’s constant) and h = 6.626 × 10^−34^ J.s (Planck’s constant). From the slope of the linear fitting, the ΔH* value for 3.0% Ni/IrSnO_2_ is equal to 20.31 kJ.mol^−1^. Additionally, from the intercept, the ΔS* value is ~−107.91 Jmol^−1^K^−1^. For comparison, the thermodynamic parameters are also estimated for SnO_2_ and SnO_2_:Ir electrodes using Figure S4 and Figure S5 (Supplementary data). For the SnO_2_ electrode, the Ea, ΔH*, and ΔS* values are ~14.308 kJ.mol^−1^, 13.685 kJ.mol^−1^, and −66.189 Jmol^−1^K^−1^, respectively. For the SnO_2_:Ir electrode, these values are changed to 16.971 kJ.mol^−1^, 23.83 kJ.mol^−1^, and −637.68 Jmol^−1^K^−1^, respectively. 

#### 3.3.4. Effect of Monochromatic Light Illumination and Conversion Efficiencies

**Figure 9:**
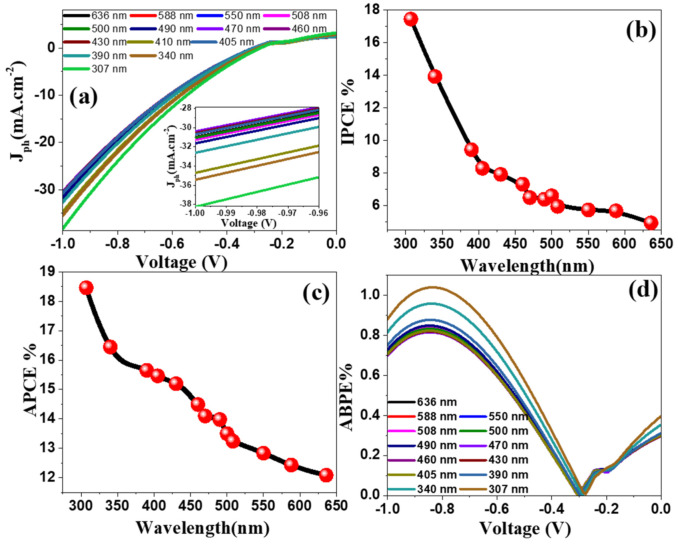
(**a**) *J_ph_*–voltage characteristics of 3.0% Ni-doped SnO_2_:Ir photoelectrode under monochromatic light illumination at RT in 0.5M HCl, (**b**) IPCE%, (**c**) ABCE% (λ) at −1 V versus the incident λ, and (**d**) ABPE% as a function of the applied V at different λ. The inset of (**a**) shows the effect of wavelength on the *J_ph_*–voltage at high potential window.

Figure 9a shows *J*_*ph*_ versus the applied voltage under the monochromatic light illumination for the 3.0% Ni-doped SnO_2_:Ir photocathode in 0.5M HCl solution at RT. A set of bandpass filters with wavelengths from 307 to 636 ± 10 nm were used. From the inset of Figure 9a, the highest photocurrent occurred at 307 nm with *J_ph_* = 38.25 mA/cm^2^. The least current was created at 470 nm with *J_ph_* = 30.41 mA cm^−2^. This *J_ph_*–wavelength dependence may be correlated with the absorption behavior of the 3.0% Ni/IrSnO_2_ photocathode at each wavelength and supports the photoelectrocatalytic response of the optimized photoelectrode for the H_2_ generation process. Generally, this behavior confirms the comprehensive response of the 3.0% Ni/IrSnO_2_ photoelectrode and its capacity to absorb a large portion of the solar spectrum in UV/visible regions.

The enhanced solar absorption of the 3.0% Ni-doped SnO_2_:Ir photoelectrode and its application for efficient H_2_ production from H_2_O splitting is further ensured by calculating the incident photon-to-current conversion efficiency (IPCE) at diverse wavelengths (λ). The IPCE% is calculated at a fixed applied voltage (−1 V) by Equation (5) [44]:

(5) $$\mathrm{IPCE}\%=1240\cdot \frac{J_{ph}}{\lambda \cdot \rho }\cdot \;100$$

where ρ is the illuminating light power density of the xenon lamp (Newport, 66142-500HX-R07, Newport, UK) as a function of the monochromatic light wavelength—see Supplementary Data, Figure S6 and Table S2. The variation of IPCE% with λ is signified in Figure 9b. The maximum IPCE% for the 3.0% Ni-doped SnO_2_:Ir photoelectrode is ~17.43% at 307 nm, which matches the maximum absorption of the optimized electrode.

The optical loss effects, including the incident photon transmission (Tr) or reflection (R), were ignored in the IPCE calculations. To measure the internal quantum efficiency, which is often called the absorbed photon-to-current conversion efficiency (APCE) to correct optical losses, APCE%, the number of PEC generated carriers which contribute to the generated photocurrent/absorbed photon is determined using Equation (6) [47]:

(6) $$\mathrm{APCE}(\lambda )=\frac{\mathrm{IPCE}\left(\lambda \right)}{A\left(\lambda \right)}=\frac{\mathrm{IPCE}\left(\lambda \right)}{1-R-T_{r}}$$

where A(λ) refers to the optical absorption. Figure 9c illustrates the variation of APCE% as a function of the incident λ. Two featured values are observed in this figure for APCE%: 18.45% at ~307 nm and 15.2% around 430 nm. These results agree well with the observation from Figure 4, whereas two absorption peaks and two optical bandgaps are observed for the 3.0% Ni/IrSnO_2_ photoelectrode.

When applying a small external voltage to the PEC cell, the added electric energy to the system must be deducted to determine electrode photocatalytic efficiency. For this purpose, ABPE% (the applied bias photon-to-current efficiency) can be used. The ABPE% values for the designed photocathode are calculated by Equation (7) [48]:

(7) $$\mathrm{ABPE}\%=J_{ph}\frac{\left(1.23-V_{app}\right)}{\rho }\times 100,$$

where 1.23 is the standard state reversible potential of H_2_O and *V_app_* is the external voltage. Figure 9d illustrates the ABPE% values versus voltage at different wavelengths. Table S3 (Supplementary Materials) shows the maximum values of ABPE% and corresponding voltage (E) at different monochromatic light levels for the H_2_ evolution reaction (HER) and the oxygen evolution reaction (OER). Figure S7 (Supplementary Materials) illustrates the ABPE% values as a function of applied voltage at different λ for OER. The maximum value of ABPE% is 1.038% at −0.839V and 307 nm for HER. Additionally, this photoelectrode shows an offset ABPE% of 0.391% at 307 nm and 0 V. This illustrates the decrease in the interfacial transport resistance and enhances the PEC performance. Moreover, two maximum ABPE% values are observed for OER (Table S3 and Figure S7): 0.433% at 0.166 V and 0.430% at 0.297 V at 307 nm. Note that the potential position of OER1 and OER2 shifts from 0.0653 to 1.66 V and from 0.351 to 0.297 V by decreasing the wavelength to 307 nm (Table S3). The high performance of the photoelectrode at relatively low potential could be advantageous for PEC cell setup. 

The benchmark efficiency, or solar to hydrogen efficiency (STH), is the ratio between the total H_2_ energy generated and the total energy of sunlight (AM 1.5 G, 0.1 W/cm^−2^). STH% can be measured utilizing Equation (8) [49]:

(8) $$\mathrm{STH}=[\;(H_{2}/S)\times \left(2.37\;\times \;10^{5}\;J/\mathrm{mol})]/[\;A\times P_{\mathrm{total}}\right]$$

where P_total_ and A are the power density of the illuminating light in mW/cm^2^, and the area of the illuminated part of the photoelectrode in cm^2^, respectively, and H_2_/S is the rate of the hydrogen moles production in mmol/s. Then, the calculated STH value is 12.72% for the 3.0% Ni-doped SnO_2_:Ir photocathode.

In this study, the optimized 3.0% Ni-doped SnO_2_:Ir photocathode showed higher *J_ph_* and conversion efficiency than previously reported SnO_2_-based PEC electrodes [50,51,52,53,54,55,56,57,58], as given in Table 2. Previous reports showed that the PEC *J_ph_* of a graphene/SnO_2_–TiO_2_ heterostructure and FTO/BiVO_4_/SnO_2_/WO_3_ was *J_ph_* = 5.6 mA·cm^–2^ at 1.23VRHE in 0.25 M Na_2_S and 0.35 M Na_2_SO_3_ and *J_ph_* = 4.15 mA·cm^–2^ at 1.23VRHE in 0.1 M KHCO_3_, respectively [50,57]. For a F-doped SnO_2_/CuWO_4_ (4 cycles) IO photocatalyst, the IPCE = 9.4% at 315 nm and 7.45% at 390 nm in 0.5 M Na_2_SO_4_ [55]. Additionally, an STH of 3.5% was reported for BiVO_4_/WO_3_/SnO_2_ triple layers [56]. The results demonstrate that the nanostructured 3.0% Ni-doped SnO_2_:Ir has high light-harvesting capabilities, high conversion efficiencies, and enhanced nanomorphological and nanostructural properties, which are highly favorable for practical applications.

### 3.4. Corrosion, Tafel Parameters, and Electrochemical Surface Area

**Figure 10:**
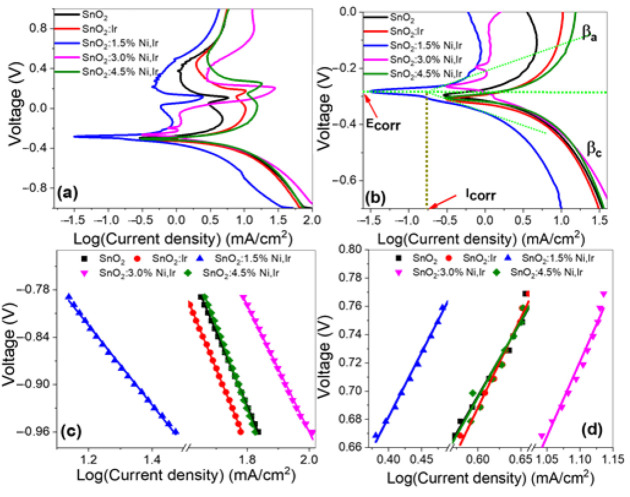
(**a**,**b**) Combined anodic and cathodic Tafel plots of all photoelectrodes provided with the characteristic parameters. Calculation of cathodic slopes ( βc ) (**c**) and anodic slopes (**d**).

The combined anodic and cathodic Tafel curves are presented based on the Tafel relation, V = β log(*J_ph_*) + c, to outline the HER mechanism and the rate-limiting phase [59]. Perfect PEC catalysts have low Tafel slopes, high current exchange rates, and therefore, good HER efficiency. However, this is not a constant rule, where some photocatalysts with higher Tafel slopes and higher current conversion rates, and vice versa, exist [60]. Figure 10a illustrates combined anodic and cathodic Tafel plots of the electrodes under analysis (potentiodynamic polarization curves). Figure 10b presents the key characteristic parameters for the electrodes; corrosion potential (E_corr_), corrosion current (I_corr_), anodic and cathodic Tafel slopes (βa and βc). For the electrodes under analysis, the βa and βc values are determined from the slopes of the linear segments of the curves, as seen in Figure 10c,d [46,61].

For all electrodes, the determined values of E_corr_, I_corr_, βa, and βc are listed in Table 3. The βa and βc values are raised by adding 3% Ir to reach 125.2 and 95.9 mV/dec and then decreased by co-doping with 1.5% Ni to 90.4 and 50.4 mV/dec, respectively, whereas their values are increased to 105.8 and 104.1 mV/dec, respectively, by increasing the Ni content to 4.5%. Tafel PEC slopes clarify what the reactions mechanisms and rate-limiting stages are in the PEC process. That is, the Volmer–Tafel mechanism is dominant when the recombination stage is rate-limiting, and the Tafel slope is ~30 mV/decade. The Volmer–Heyrovsky hydrogen evolution mechanism can be presumed dominant when PEC desorption is rate-limiting, and the Tafel slope is ~40 mV/decade. If the slope of the Tafel is ~120 mV/decade, the reaction paths depend on the surface imbued with adsorbed hydrogen. The βc value demonstrates the over-potential needed to boost the hydrogen generation reaction (HGR) rate by a factor of 10 [46,61]. For the photoelectrodes under study, low Tafel slopes of the co-doped photoelectrodes and the low energy of the bandgaps resulted in low overpotentials. This is owing to the small amount of energy needed to improve the HGR performance and vice versa. Hence, the best values of βc and βa are obtained up to 3% co-doping. This explains the high PEC performance of our photoelectrode compared to the other photoelectrodes. 

The information about the solution’s corrosion tendency can be obtained from E_corr_, whereas the corrosion rate is directly proportional to I_corr_. It can be observed from Table 3 that the 3.0% Ni-doped SnO_2_:Ir photoelectrode showed better behavior with an E_corr_ of −265 mV and an electrochemical surface area (ECSA) of 65.10 m^2^/g. The 3.0% Ni-doped SnO_2_:Ir has a higher E_corr_ (311 mV) in comparison with the 1.5% Ni-doped SnO_2_:Ir and 3.0% Ni-doped SnO_2_:Ir. Relative to the pure SnO_2_, the corrosion potential of 3.0% Ni-doped SnO_2_:Ir is moved towards the positive direction by 31 mV. Generally, the reported E_corr_ values are shifted to better behavior compared to that of the commercial SnO_2_. Moreover, they are more positive than any reported data for photoelectrodes based on SnO_2_ [62,63]. 

The values of I_corr_, corrosion rate (CR), and polarization resistance (RP) can be used to determine the photoelectrode’s relative capability to resist corrosion. The CR is directly proportional to the kinetic value of I_corr_, and Rp is inversely proportional to the kinetic value of I_corr_. As seen from Table 3, the incorporation of 3% Ir decreases the I_corr_ of the pure SnO_2_ from 359.6 to 323.6 μA/cm^2^. Additionally, the insertion of 1.5% Ni decreases the I_corr_ of SnO_2_:Ir to 98.5 μA·cm^−2^. The increment of Ni content, however, increased the I_corr_ to 318.0 μA·cm^−2^ at 3% Ni, but this is still much smaller than the corrosion current of pure photoelectrodes. The polarization resistance (Rp in Ohm/cm^−2^) was estimated from the relation; Rp = βc βa / [2.303 I_corr_ (βc + βa)] utilizing the Stern–Geary equation and the straight segment of the curves, close to E_corr_. CR was also determined (in nm/year) using CR = 3272 I_corr_[EW/d.A], where d, A and EW are the density (g/cm^3^), area (cm^2^), and the equivalent weight (g/eq), respectively. For all of our electrodes, the determined values of CR and Rp are given in Table 3. The value of Rp increases from 58.90 to 142.82 Ω.cm^2^ and CR is reduced from 6.18 to 1.69 nm/year by incorporating 1.5% Ni/3% Ir to the pure SnO_2_ photoelectrode. By increasing the co-doping ratio to 4.5%, the CR is raised marginally to 5.35 nm/year. The values of the corrosion parameters mentioned indicate the considerable enhancement of the corrosion resistance through the implementation of optimized Ni/Ir co-doping into the photoelectrodes. This further demonstrates the essential role of the Ni/Ir co-doping in improving the electrode’s stability. Our obtained values of CR are better than any previously reported SnO_2_-based photoelectrode values [64,65]. Therefore, the corrosion parameters in Table 3 highlight the corrosion inhibition effects of 1.5% Ni-doped SnO_2_:Ir, and the Tafel slopes of this photoelectrode also demonstrate a decrease in system dissolution reactions. This can be attributed to the more compact structure at this co-doping ratio due to the decrease in grain size and crystallite size and the development of a high density of smaller nanocrystals (XRD), in addition to the decrease in R_rms_, which decreases the scattering of the carrier inside the electrode and electrochemical surface area and thus hinders corrosion.. This can thus be attributed to a more compact structure, a lower porosity, and a smoother surface. However, the increase in Ni to 3% changes the surface morphology of SnO_2_:Ir from nanocrystals islands with heights ≤ 10 nm to a 1D tubular nanostructure, which is attractive for solar conversion by offering direct pathways for the charge carrier transport, the highest electrochemical surface area (65.1 m^2^/g), and the lowest optical band gap (2.70 eV) due to the insertion of electronic states in the bulk SnO_2_ and co-doping-related oxygen defects. 

The electrochemical surface areas (ECSAs) of the prepared photocatalytic electrodes are obtained using the Randles–Sevcik relation, ECSA = I(Rg T)1/2 (Ca N Fc)−3/2(v Da)-1/2/0.4463, wherever Rg, Ca, Fc, Da, and N indicate to the gas-molar constant, analyte concentration, and number of the electrons in the redox reaction (N = 1 in this study), respectively [46]. Utilizing Figure 5a, the ECSAs of the electrodes under analysis are calculated from ECSA = QH. mc^−1^. Cm^−1^, where QH, Cm, and mc are the negative-scan-hydrogen-adsorption charges after double-layered charge adjustment, the full charge of electrode-covering monolayer H-atoms, and catalyst mass, respectively [46]. The values of QH are determined from the integration of each photoelectrode’s curve divided by the measurement-scanning rate (10 mV) in Figure 5a. The obtained ECSAs are illustrated in Table 3. For the pure and 3.0% Ni/IrSnO_2_, the ECSA values are 44.69 and 65.1 m^2^ g^−1^, respectively. The high ECSA indicate the high PEC performance of the 3.0% Ni-doped SnO_2_:Ir photoelectrode compared to the other photoelectrodes [61].

### 3.5. PEC Impedance Spectroscopy (PEC-IS)

**Figure 11:**
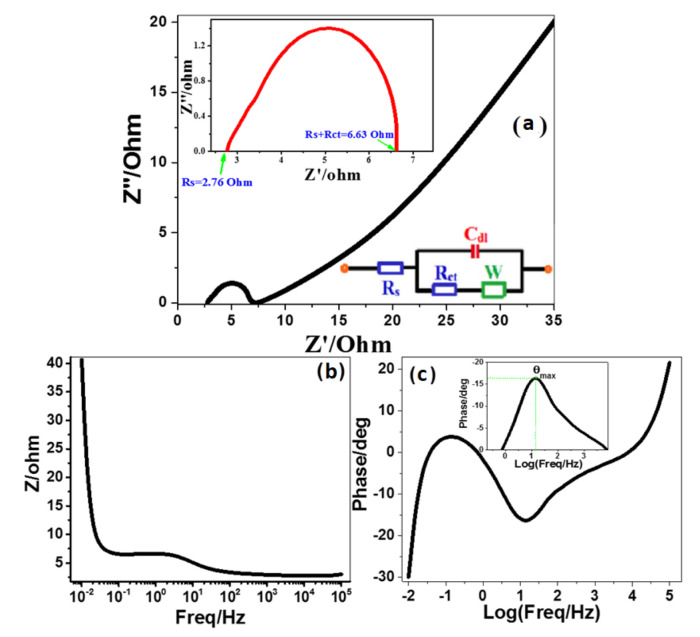
Nyquist plot (**a**) and Bode plots for 3.0% Ni/IrSnO_2_ photoelectrode in 0.5 M HCl electrolyte at 25 °C and 0 V (vs. Ag/AgCl) and under visible light illumination; (**b**) the variation of the total impedance with frequency and (**c**) the variation of phase with frequency. The inset circuit of (**a**) represents a simple Randle equivalent circuit. The inset of (**c**) shows the value of θ_max_.

The impedance of the photoelectrochemical system depends on the charge transfer between the active electrode and the electrolyte interface. PEC-IS measurements were carried out at RT utilizing an electrochemical workstation (CH Instruments CHI660E) to examine the charge carrier dynamics of the optimized 3.0% Ni-doped SnO_2_:Ir photoelectrode. The PEC-IS measurements were performed in a frequency (f) range of 10^−2^–10^5^ Hz at 0 V (vs. Ag/AgCl) under illumination. Figure 11a shows a Nyquist plot of 3.0% Ni/IrSnO_2_ immersed in 0.5M HCl. The obtained results are also presented in Bode plots (Figure 11b,c). The Nyquist plot of 3.0% Ni-doped SnO_2_:Ir photoelectrode shows that there is a small semicircle with a small diameter in the high-f side, indicating the redox reaction [66], followed by a linear stage covering the region of middle/low fs. These PEC-IS spectra displayed mixed diffusion/kinetic controlled routes. To understand the PEC-IS measurements through HER (Figure 11), the obtained spectra are fitted to a simple Randle equivalent circuit (REC). Figure 11a’s inset displays the REC that was used to model the PEC-IS spectra utilizing the ZSimpWin software. This equivalent circuit consists of electrolyte resistance (Rs = 2.76 Ω) obtainable at high fs from Nyquist plot intercepts, charge transfer resistance (R_ct_ = 3.87 Ω) equal to the semicircle diameter in the Nyquist plot, double-layer capacitance (C_dl_ = 19.63 mF) and Warburg impedance (W = 0.9). Then, the charge transfer process (CTP) is the main regulator of the HER, which is guaranteed by the uni-loop of the Nyquist plot. Although R_ct_ shows a higher value relative to the other elements of the equivalent circuit, this R_ct_ value is much smaller than most of the literature values for SnO_2_-based PEC electrodes [55,67]. For example, F-doped SnO_2_ IO and F-doped SnO_2_/CuWO_4_ IO showed significantly higher R_ct_ values of 18890 Ω and 8000–10000 Ω, respectively [55]. To the best of our knowledge, only the Ti/IrO_2_/Sb-SnO_2_ electrode showed an R_ct_ value of the same order (1.65 Ω), but with much smaller J_ph_ than our 3.0% Ni-doped SnO_2_:Ir electrode [58]. The small value of R_ct_, which represents the kinetic or charge transfer resistance to the ion transfer and is equal to the diameter of the depressed capacitive semicircle in the inset of Figure 11a, refers to enhanced ionic conduction and electrolyte diffusion over the electrode nanoporous structure [68]. Then, the optimized electrode can produce a large amount of H_2_. Besides the charge transfer process (CTP), the e/h recombination is mostly used to control the HER. The reordered value of R_ct_ is very small, which means that the charge recombination on the electrode/electrolyte interface was highly reduced, which supports the improved HET [69]. 

Figure 11b,c shows Bode plots for the photocathode at the H_2_ evolution potential in 0.5 M HCl aqueous liquid at 25 °C. Figure 11b shows the variation of the total impedance with f, and Figure 11c illustrates the variation of phase with f. This figure shows resistive regimes at low and very high frequencies, in addition to capacitive contribution in between. The low-frequency regime is correlated with the charge transfer resistance (R_ct_) and the double-layered capacitance (C_dl_) of the electrode. The very high-f regime may be related to the formation of a partially protective layer on the surface of the electrode. The maximum phase shift (θ_max_= 16.4°) is detected at 14.68 Hz. The lifetime of the charge carriers can be estimated from Figure 11c by the relation $\tau_{n}$ = 1/2π ƒmax [70]. The calculated lifetime for the optimized electrode is 10.85 ms. This value is almost 31 times the reported value for pure SnO_2_ (0.35 ms) [71]. Then, the determined parameters from Figure 11 indicate a great reduction in the charge recombination at the electrolyte/photoelectrode interfaces. This also refers to a kinetically facile PEC system, improved ionic conductivity, and electrolyte diffusion through the 3.0% Ni-doped SnO_2_:Ir photoelectrode.

### 3.6. Sample Purity after Photoelectrochemical Measurements

**Figure 12:**
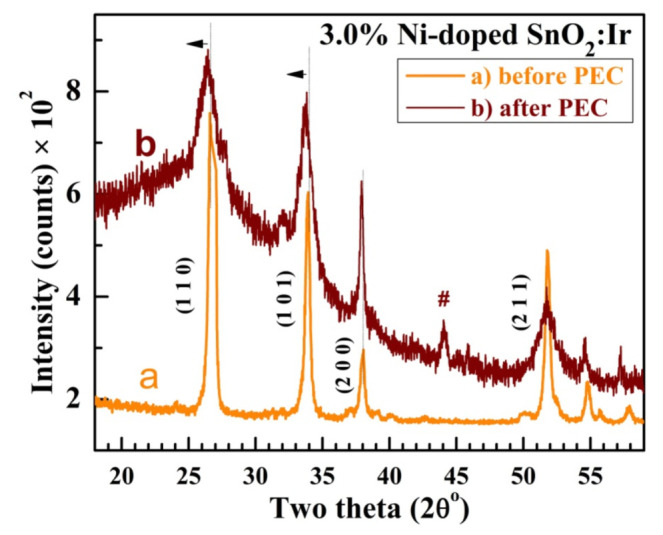
XRD patterns of the 3.0% Ni-doped SnO2:Ir before and after PEC measurements. The arrows (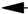) shows the shift of peaks peaks after PEC and (#) denotes to unknown peak.

XRD was measured for the optimized electrode, 3.0% Ni-doped SnO_2_:Ir, after PEC measurements and is presented in Figure 12 relative to its XRD chart before PEC measurement. As seen, the (1 1 0), (101), and (2 0 0) peaks are slightly shifted to lower 2θ angles, indicating a marginal increase in d-spacing of the sample. The diffraction peaks became broader with reduced intensity, except for (2 0 0). The *D*_av_ decreased to 18.2 nm after PEC measurements. Only one small unknown peak is observed at 2θ = 44.03°. This result illustrates the high purity of the 3.0% Ni-doped SnO2:Ir photoelectrode.

## 4. Conclusions

SnO_2_, SnO_2_:Ir, and (1.5–4.5%) Ni-doped SnO_2_:Ir films were deposited on a glass substrate using the spin-coating method. The results of XRD illustrate that all samples are polycrystalline structures with a rutile phase. The peak shift in XRD patterns and FTIR spectra confirmed the formation of homogenous composites. XRD and AFM showed that the films were compact, had granular structures, and that D (and GS) decreased from 30.43 nm (33.12 nm) to 24.23 nm (23.81 nm) with increasing Ni loading. Additionally, R_rms_ decreased from 17.25 to 11.64 nm. Ni-doped films show an absorption band at ~255 nm that shifted to lower wavelengths for pure and SnO_2_:Ir films. Additionally, the direct *E_g_* blue-shifted to 3.70 eV at 3.0% Ir doping, and then red-shifted to 2.7 eV for the Sn_0.94_Ir_0.03_Ni_0.03_O_2_ composition. The tendency of these films to form Sn–OH bonds, the decrease in R_rms_, and *E_g_* red-shifting to 2.7 eV are encouraging factors for H_2_ generation from water. The key PEC factors for performance for pure, 3% Ir-doped, and Ni/Ir-co-doped electrodes were explored and improved for effective H_2_ generation utilizing sunlight. Amongst the investigated PEC electrodes, the 3.0% Ni-doped SnO_2_:Ir displays the highest photocurrent density of 46.38 mA/cm^2^ and the highest PEC H_2_ generation rate of 52.22 mmol h^−1^cm^−2^ at −1V with an IPCE% of ~17.43% at 307 nm. Additionally, this electrode showed an ABPE of 1.038% at −0.839 V and a surprisingly offset value of 0.391% at 0 V and 307 nm for HER, which are the highest values yet for SnO_2_-based PEC catalysts. Moreover, this photoelectrode displays a photogenerated current density of −432 μA/cm^2^ at 0 V and photocurrent onset over −0.196 V. The effect of the electrolyte type was studied, the current density was ranked as follows: *J_ph_*(HCl) > *J_ph_*(NaOH) > *J_ph_*(Na_2_SO_4_). The βa and βc values are raised by adding 3% Ir to reach 125.2 and 95.9 mV/dec and then decreased by co-doping with 1.5% Ni to 90.4 and 50.4 mV/dec, respectively. As the T of the PEC process increases to 85 °C, the current density displays ~3.5-fold enhancement.

The thermodynamic factors have been obtained using the 3.0% Ni-doped SnO_2_:Ir electrode; activation energy= 17.598 kJ/mol, entropy =107.91 J/mol^.^K, and enthalpy = 20.31 kJ/mol. Additionally, this electrode demonstrated a higher electrochemical surface area (~ 1.8 times) and lower Tafel slopes (103.0 and 70.26 mv/dec) relative to the 3% Ir-doped photoelectrode. Moreover, the rate of corrosion changes from 6.18 to 1.69 nm year^−1^ with the incorporation of 1.5% Ni/3% Ir co-dopants in the pure SnO_2_ electrode. All of the key parameters demonstrated a significant decrease in charge recombination at the electrode/electrolyte interface, which was used to pinpoint the PEC H_2_ generation mechanism.. From the stability study and after 12 runs of reusability at −1 V, the optimized photoelectrode preserved ~94.95% of its initial PEC performance. This work provided a new doping strategy to develop a new collection of highly active SnO_2_-based photoelectrodes for sustainable PEC hydrogen fuel production under sunlight illumination.

## Figures and Tables

**Table 1:** Films’ thicknesses, XRD data (lattice parameters a and c and volume of the unit cell V), and crystallite size *D*_av_. AFM measurements (grain size, Gs, and roughness, R_rms_) and the direct optical bandgap (*Eg*) of the films) are also shown.

Film’s Composition	Thickness (nm)	Lattice Parameter			*D*_av_ (nm)	AFM		*E*_g_ (eV)
		*a* (Å)	*c* (Å)	*V* (Å^3^)		Gs (nm)	R_rms_ (nm)	
SnO_2_	222	4.747	3.189	71.86	29.62	33.12	17.25	3.50
SnO_2_:Ir	219	4.742	3.191	71.75	30.43	35.07	15.36	3.70
SnO_2_: 1.5% Ni,Ir	225	4.741	3.182	71.52	27.57	29.37	19.17	2.77
SnO_2_: 3.0% Ni,Ir	217	4.735	3.178	71.25	23.91	26.92	14.23	2.70
SnO_2_: 4.5% Ni,Ir	215	4.712	3.173	70.45	24.23	23.81	11.64	2.75

**Table 2:** Values of *J_ph_* and conversion efficiency of the optimized 3.0% Ni/IrSnO_2_ photoelectrode relative to the previously reported SnO_2_-based PEC electrodes for H_2_O splitting.

Photoelectrode Photoelectrode	Electrolyte	PEC Performance	Ref
3.0% Ni-doped SnO_2_:Ir	0.5M HCl	*J_ph_* = 46.38 mA·cm^−2^ at −1V, IPCE = 17.43% and APCE% = 18.46% at 307 nm, 52.22 mmol h^−1^ cm^−2^ at −1 V	This work
SnO2-TiO_2_ heterostructure	0.25 M Na_2_S and 0.35 M Na_2_SO_3_ (pH ∼13)	*J_ph_* = 4.7 mA/cm^2^	[50]
Graphene/ SnO_2_–TiO_2_ heterostructure		*J_ph_* = 5.6 mA cm^−2^	
Pt/SnO_2_/RuO_2_	0.1 M H_2_SO_4_	Rate of H_2_ production = 3.75 × 10^−5^ mol dm^−3^	[51]
FTO/SnO_2_ /W:BiVO_4_	0.15 M K_2_SO_4_ (pH 7)	1.4 mA/cm²	[52]
FTO/SnO_2_/W:BiVO_4_ /Co-Pi	0.1 M KPi (pH 7)	2.3 mA/cm²	[53,54]
Fluorine-Doped SnO_2_ Inverse Opal(IO)	0.5 M Na_2_SO_4_	*J_ph_* = 0.14 mA/cm^2^ at 1.23 VRHE, IPCE = 3.42% at 315 nm	[55]
F-Doped SnO_2_ /CuWO_4_ (4 cycles) IO film		*J_ph_* = 0.42 mA/cm^2^ at 1.23 VRHE, IPCE = 9.4% at 315 nm and 7.45% at 390 nm	
BiVO_4_/WO_3_/SnO_2_ triple layers	0.5 M phosphate buffer electrolyte (pH 7)	*J_ph_* = 3.1 mA/cm^2^ at 1.23 VRHE, STH = 3.5%	[56]
FTO/BiVO_4_ /SnO_2_ /WO_3_ (double stacked)	0.1 M KHCO_3_ (pH 8)	*J_ph_* = 4.15 mA/cm^2^ at 1.23 VRHE	[57]
Ti/IrO_2_/Sb-SnO_2_ electrode	0.5 M Na_2_SO_4_	*J_ph_* = 0.4 mA/cm^2^ at 3 V	[58]

**Table 3:** Values of corrosion and Tafel parameters, and ECSA of our photoelectrodes.

Sample	E_Corr_ (mV)	I_Corr_ (μA cm^−2^)	βa (mV dec^−1^)	R^2^	Βc (mV dec^−1^)	R^2^	R_p_ (Ohm/cm^−2^)	Corr Rate (nm year^−1^)	ECSA (m^2^ g^−1^)
SnO_2_	−296	480.84	102.1 ± 4.4	0.9999	93.16 ± 0.23	0.9723	58.90	6.18	44.69
SnO_2_:Ir	−311	527.23	125.2 ± 5.3	0.9993	95.86 ± 0.64	0.9851	72.94	5.56	36.90
1.5% Ni doping	−282	163.29	90.4 ± 2.4	0.9983	50.35 ± 0.52	0.9943	142.82	1.69	16.02
3.0% Ni doping	−265	394.91	103.0 ± 6.1	0.9987	70.26 ± 1.03	0.9682	57.11	5.46	65.10
4.5% Ni doping	−306	517.13	105.8 ± 8.4	0.9995	104.09 ± 0.57	0.9530	73.31	5.35	41.34

## Data Availability

Not applicable.

## Supplementary Materials

The following supporting information can be downloaded at: https://www.mdpi.com/article/10.3390/nano12030453/s1, Table S1: The current density and voltage-position of the anodic peaks I and II in HCl electrolyte; Table S2: light power intensity of Xenon lamp at different monochromatic wavelength; Table S3: Maximum Values of ABPE% and corresponding voltage (E) at different monochromatic lights for HER and OER; Figure S1. Cross-sectional investigation for (a) SnO_2_, (b) SnO_2_:Ir, and (c) SnO_2_: 4.5% Ni, Ir films; Figure S2: Photocurrent intensity for of 3.0%Ni/IrSnO2 photocathode under successive on/off illumination cycles, measured in 0.5 M HCl electrolyte under a bias potential of −1V. The electrode’s holding time in the light is 35 s; Figure S3: Long-term stability test; photocurrent intensity for of 3.0%Ni-doped SnO_2_:Ir electrode under successive on/off illumination cycles in 0.5 M HCl electrolyte @ −1V; Figure S4: Effect of temperature on SnO_2_; (a) the Jph-voltage plots at temperatures in the range of RT −85 °C, (b) ln(Jph) & (1/T), and (c) Ln (Jph/T) & (1/T) polts; Figure S5: Effect of temperature on SnO_2_:Ir; (a) the Jph-voltage plots at temperatures in the range of RT −85 °C, (b) ln(Jph) & (1/T), and (c) Ln (Jph/T) & (1/T) polts; Figure S6: Spectrum of Xenon lamp; Figure S7: ABPE% for of 3.0%Ni/IrSnO_2_ photocathode under different monochromatic illumination in the anodic region.
