# Correction: Shaban et al. Design of SnO_2_:Ni,Ir Nanoparticulate Photoelectrodes for Efficient Photoelectrochemical Water Splitting. *Nanomaterials* 2022, *12*, 453

**DOI:** 10.3390/nano15191490

**Published:** 2025-09-29

**Authors:** Mohamed Shaban, Abdullah Almohammedi, Rana Saad, Adel M. El Sayed

**Affiliations:** 1 Department of Physics, Faculty of Science, Islamic University of Madinah, Al-Madinah Al-Munawarah 42351, Saudi Arabia; ard.almohammedi@hotmail.com; 2 Nanophotonics and Applications (NPA) Lab, Physics Department, Faculty of Science, Beni-Suef University, Beni-Suef 62514, Egypt; ranasaad811@gmail.com; 3 Department of Physics, Faculty of Science, Fayoum University, El-Fayoum 63514, Egypt; adel_sayed_2020@yahoo.com

In the original publication [1], there was a mistake in Figure 1 as published. The corrected Figure 1 appears below. The legend for Figure 1 has been updated accordingly.

The “Islamic University in Madinah” in affiliation 1 has been updated to “Islamic University of Madinah”, and the corresponding author’s email address has been updated to mssfadel@iu.edu.sa.

The authors state that the scientific conclusions are unaffected. This correction was approved by the Academic Editor. The original publication has also been updated.

## Figures and Tables

**Figure 1:**
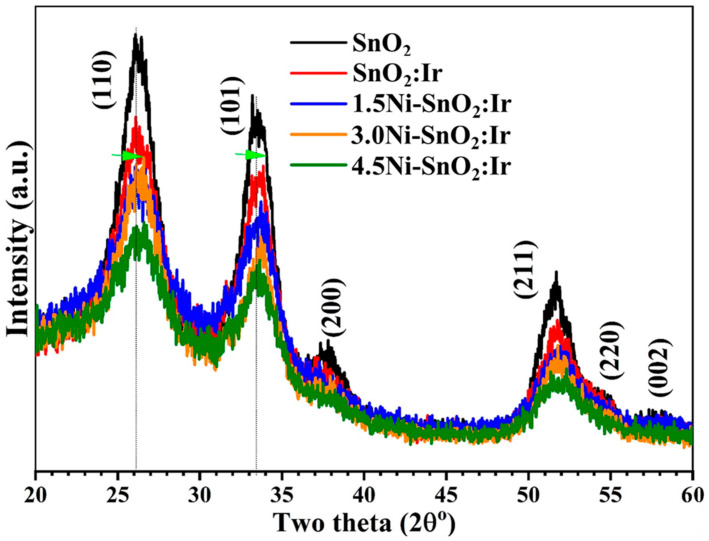
XRD patterns of SnO_2_, 3.0% Ir-doped SnO_2_ and SnO_2_:Ni,Ir thin films. The arrows indicate the peak shift.
