# Supplementary material for: Design of SnO2:Ni,Ir Nanoparticulate Photoelectrodes for Efficient Photoelectrochemical Water Splitting
Source: Nanomaterials (Basel). 2022 Jan 28;12(3):453. doi: 10.3390/nano12030453 (PMC8839913; doi:10.3390/nano12030453)
Supplement: Supplementary file 1 [file nanomaterials-12-00453-s001.zip › nanomaterials-1545166-supplementary-proof done.pdf]

nanomaterials

Supplementary Materials
Design of SnO_2:Ni,Ir Nanoparticulate Photoelectrodes for Efficient Photoelectrochemical Water Splitting

Mohamed Shaban [1,2,*], Abdullah Almohammedi [1], Rana Saad [2] and Adel M. El Sayed [3]

1 Department of Physics, Faculty of Science, Islamic University in Madinah, Al-Madinah Al-Munawarah 42351, Saudi Arabia; ard.almohammedi@hotmail.com
2 Nanophotonics and Applications (NPA) Lab, Physics Department, Faculty of Science, Beni-Suef University, Beni-Suef 62514, Egypt; ranasaad811@gmail.com
3 Department of Physics, Faculty of Science, Fayoum University, Fayoum 63514, Egypt; adel_sayed_2020@yahoo.com
* Correspondence: mssfadel@aucegypt.edu

Table S1. The current density and voltage-position of the anodic peaks I and II in HCl electrolyte.

Sample	Peak I		Peak II	
	E (mV)	J (mA/cm^2)	E (mV)	J (mA/cm^2)
SnO_2	106.7	1.57	−108.8	1.30
3%$IrSnO_2$	176.0	2.84	−9.4	2.84
1.5%Ni/$IrSnO_2$	116.4	0.69	-	-
3.0%Ni/$IrSnO_2$	203.8	7.65	-	-
4.5%Ni/$IrSnO_2$	249.8	4.89	25.2	4.19

Table S2. light power intensity of Xenon lamp at different monochromatic wavelength:.

Wavelength(nm)	P(mW/cm^2)
307	99.65
390	78.75
405	75.96
460	69.36
470	69.36
490	69.36
500	69.36
508	69.11
588	74.01

Table S3. Maximum Values of ABPE% and corresponding voltage (E) at different monochromatic lights for HER and OER.

Wavelength (nm)	HER			OER			
				OER1		OER2	
	E(V)	ABPE%	Offset ABPE%@0V	E(V)	ABPE%	E(V)	ABPE%
636	−0.84916	0.8296	0.3022	0.06534	0.3094	0.34683	0.3152
588	−0.84916	0.8273	0.3023	0.06534	0.3053	0.35131	0.3115
550	−0.84916	0.8289	0.3023	0.06534	0.3052	0.35131	0.3118
508	−0.84916	0.8323	0.3024	0.0845	0.3051	0.35131	0.3124
500	−0.84916	0.8262	0.2973	0.0845	0.2989	0.35131	0.3067
490	−0.84916	0.8404	0.3045	0.08971	0.3061	0.35131	0.3135

470	−0.84916	0.8055	0.2970	0.08971	0.2953	0.35131	0.3020
460	−0.84916	0.8114	0.2933	0.08971	0.2953	0.35131	0.3039
430	−0.84916	0.8140	0.2958	0.08971	0.2976	0.35131	0.3056
405	−0.84916	0.8166	0.2982	0.0845	0.3000	0.35131	0.3072
390	−0.84916	0.8749	0.3077	0.12571	0.3191	0.34683	0.3266
340	−0.83921	0.9550	0.3494	0.16606	0.3747	0.31673	0.3765
307	−0.83921	1.0381	0.3912	0.16606	0.4335	0.2966	0.4309

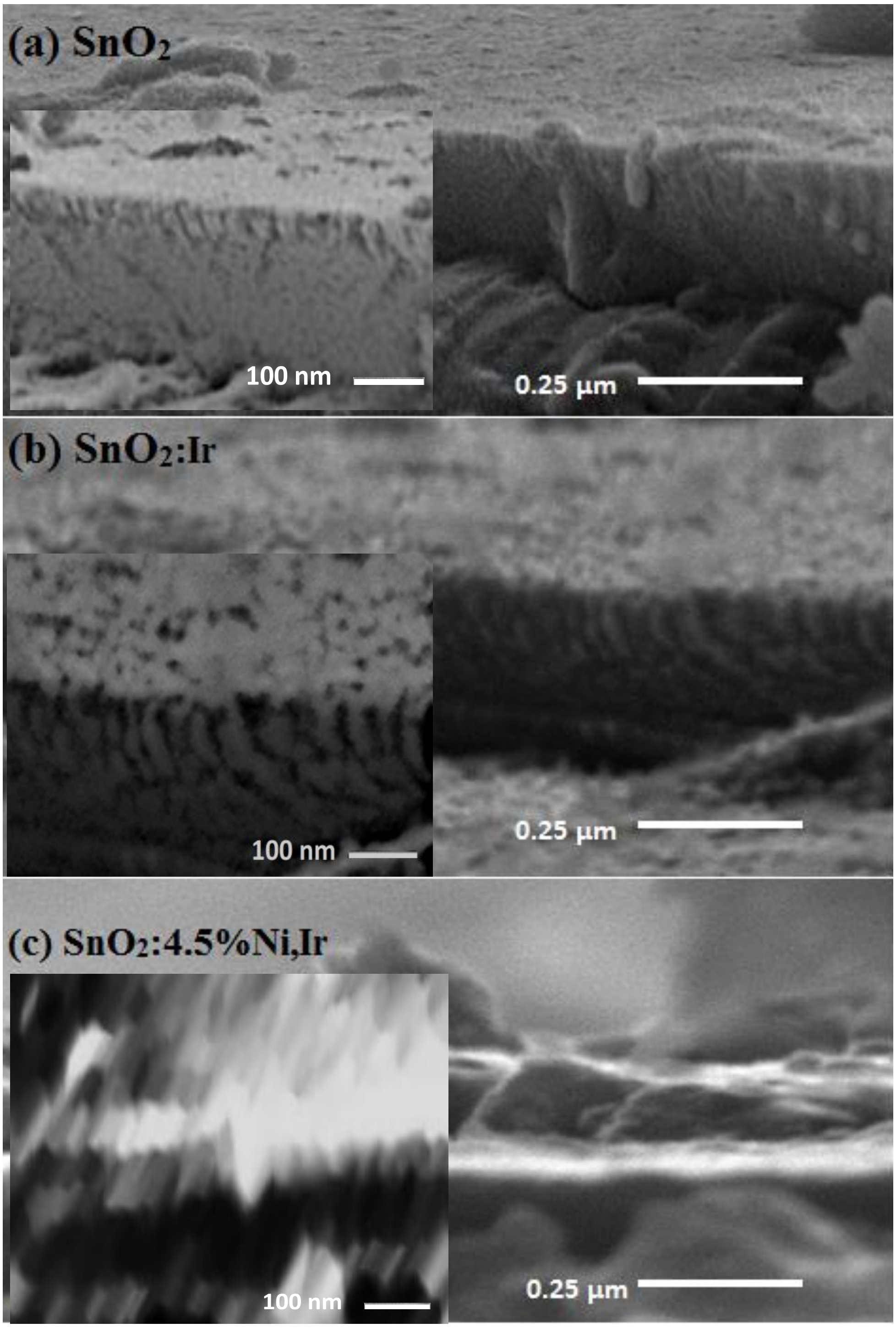

Figure S1. Cross-sectional investigation for (**a**) SnO_2, (**b**) SnO_2:Ir, and (**c**) SnO_2: 4.5% Ni, Ir films.

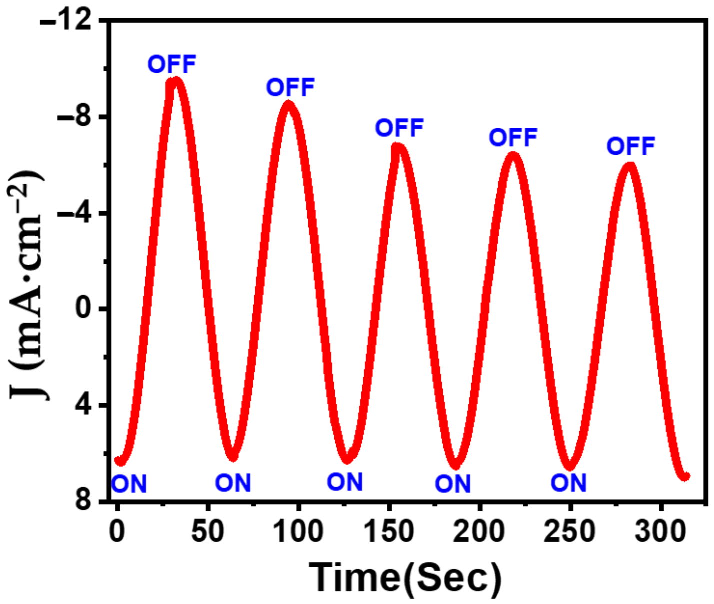

Figure S2. Photocurrent intensity for of 3.0%Ni/IrSnO2 photocathode under successive on/off illumination cycles, measured in 0.5M HCl electrolyte under a bias potential of −1V. The electrode's holding time in the light is 35 sec.

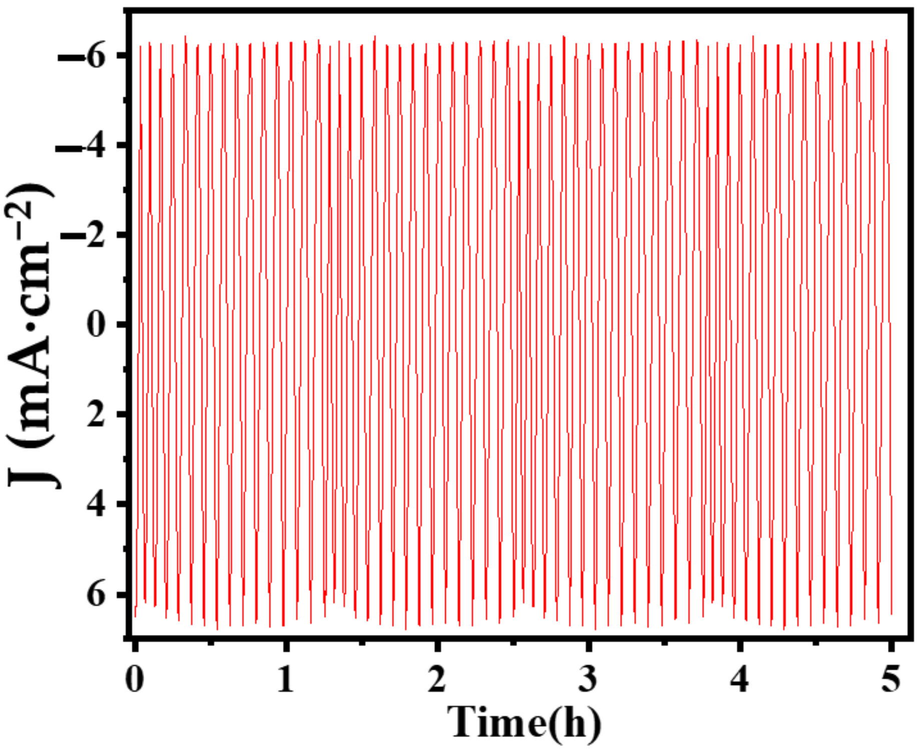

Figure S3. Long-term stability test; photocurrent intensity for of 3.0%Ni-doped SnO2:Ir electrode under successive on/off illumination cycles in 0.5M HCl electrolyte @ −1V.

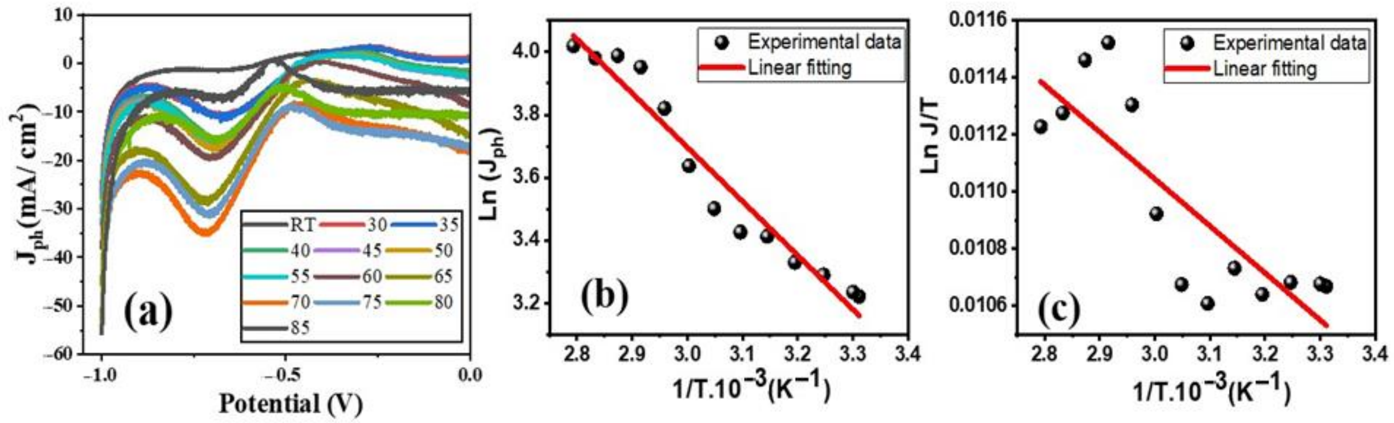

Figure S4. Effect of temperature on SnO_2; (**a**) the J_{ph}-voltage plots at temperatures in the range from RT to 85 °C, (**b**) ln(J_{ph}) & (1/T), and (**c**) Ln (J_{ph}/T) & (1/T) polts.

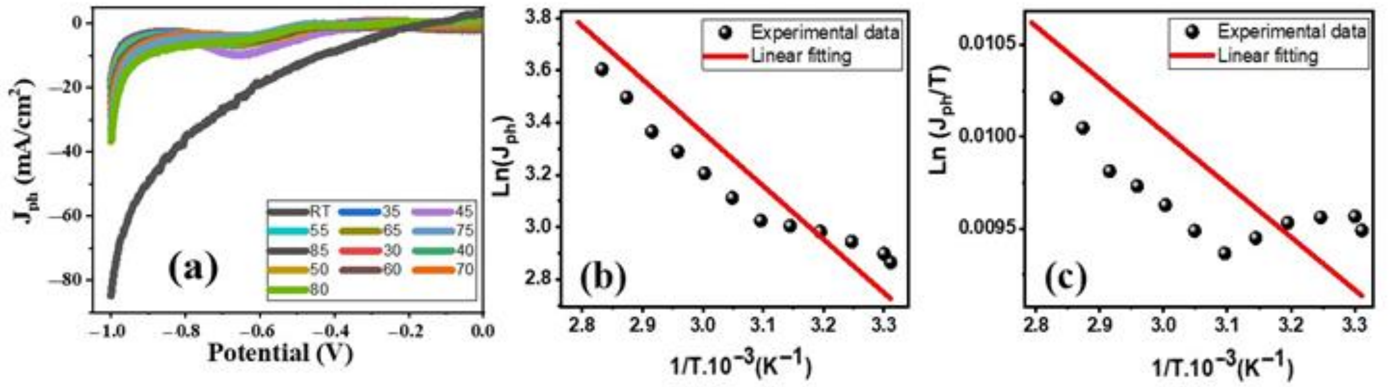

Figure S5. Effect of temperature on SnO_2:Ir; (**a**) the J_{ph}-voltage plots at temperatures in the range from RT to 85 °C, (**b**) ln(J_{ph}) & (1/T), and (**c**) Ln (J_{ph}/T) & (1/T) polts.

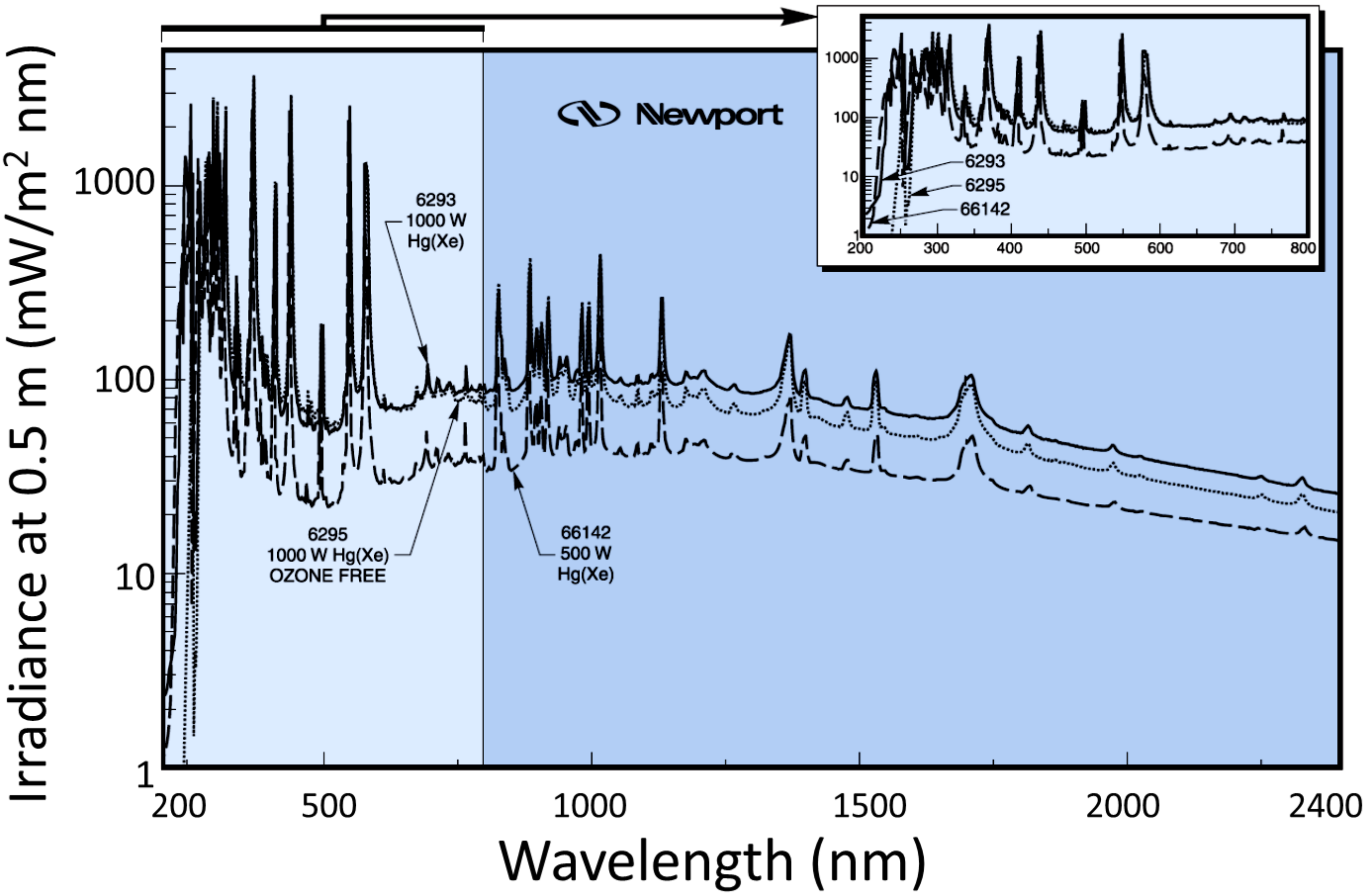

Figure S6. Spectrum of Xenon lamp.

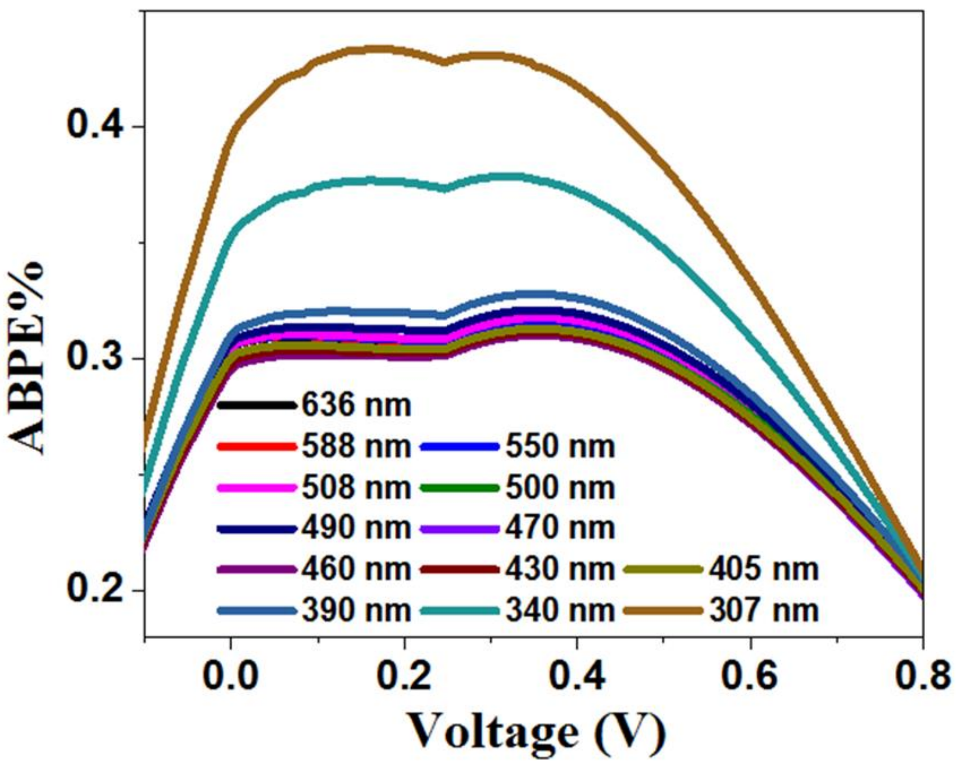

Figure S7. ABPE% for of 3.0%Ni/IrSnO₂ photocathode under different monochromatic illumination in the anodic region.
